# Formation and Reactivity of a Fleeting Ni^III^ Bisphenoxyl Diradical Species

**DOI:** 10.1002/anie.202211345

**Published:** 2022-09-02

**Authors:** Ayushi Awasthi, Isaac F. Leach, Silène Engbers, Rakesh Kumar, Raju Eerlapally, Sikha Gupta, Johannes E. M. N. Klein, Apparao Draksharapu

**Affiliations:** ^1^ Southern Laboratories-208 A Department of Chemistry Indian Institute of Technology Kanpur Kanpur 208016 India; ^2^ Molecular Inorganic Chemistry Stratingh Institute for Chemistry University of Groningen 9747 AG Groningen The Netherlands

**Keywords:** High-Valent Nickel Species, Oxidation States, Redox-Active Ligands, Salen Ligands

## Abstract

Cytochrome P450s and Galactose Oxidases exploit redox active ligands to form reactive high valent intermediates for oxidation reactions. This strategy works well for the late 3d metals where accessing high valent states is rather challenging. Herein, we report the oxidation of Ni^II^(salen) (salen=*N*,*N′*‐bis(3,5‐di‐*tert*‐butyl‐salicylidene)‐1,2‐cyclohexane‐(1*R*,2*R*)‐diamine) with *m*CPBA (meta‐chloroperoxybenzoic acid) to form a fleeting Ni^III^ bisphenoxyl diradical species, in CH_3_CN and CH_2_Cl_2_ at −40 °C. Electrochemical and spectroscopic analyses using UV/Vis, EPR, and resonance Raman spectroscopies revealed oxidation events both on the ligand and the metal centre to yield a Ni^III^ bisphenoxyl diradical species. DFT calculations found the electronic structure of the ligand and the d‐configuration of the metal center to be consistent with a Ni^III^ bisphenoxyl diradical species. This three electron oxidized species can perform hydrogen atom abstraction and oxygen atom transfer reactions.

## Graphical abstract

Ni^II^(salen) complexes have been one of the most promising avenues for those aiming to generate high valent Ni species. The present work is a result of one such inquisitiveness where a Ni^II^(salen) complex was treated with *m*CPBA to form a novel Ni^III^ bisphenoxyl diradical species. Various spectroscopic tools and theoretical calculations further assisted our observation. This proposed three electron oxidized species showed oxidation reactivity.

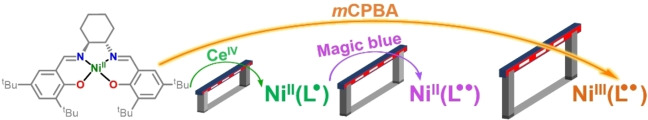

## Introduction

**Figure 1:**
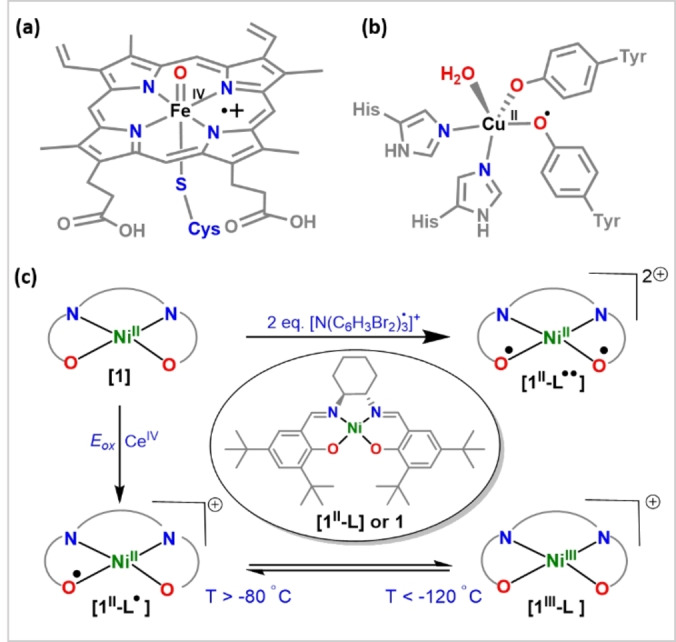
Metalloenzymes with redox active ligands: Compound I in heme‐containing a) cytochrome P450s and b) galactose oxidase (GO). c) Scheme depicting oxidation species known with **1** to date. *E*_ox_—Electrochemical oxidation.

The efficacy of the various metalloenzymes as biological catalysts depends upon the interaction between the metal and its chemical environment.^[1]^ Traditionally, ligands play a spectator role, with the metal being the center of reactivity. However, advancements have established the use of redox‐active ligands that can participate in redox events to form radical species.^[2]^ Such systems where both ligand and metal centers contribute to the redox properties of the complex have attracted considerable interest over the past decade since they can facilitate vital multi‐electron transformations that are otherwise difficult to achieve.^[3]^ For instance, Compound I in heme‐containing cytochrome P450s (CYPs), Figure 1a, responsible for hydroxylation of C−H bonds, is a Fe^IV^=O species with an additional oxidizing equivalent resonating over the porphyrin and thiolate ligands in the form of a radical.^[4]^ The copper containing enzyme galactose oxidase (GO), Figure 1b, catalyzes the two‐electron oxidation of primary alcohols to aldehydes and subsequently reduces dioxygen to hydrogen peroxide. Both a single electron transition from the Cu^I^/Cu^II^ redox couple and the tyrosine radical cross‐linked to cysteine in its ligand framework provide the necessary two oxidizing equivalents.[^1^, ^2^, ^3^, ^5^]

More broadly, numerous bioinspired routes have been investigated to gain an in‐depth understanding of the enzymatic systems. Such approaches are often used to generate high valent late 3d‐metal species.^[6]^ High valent Ni species are often proposed to be key intermediates in oxidation^[7]^ and coupling^[8]^ reactions. For example, spectroscopic and reactivity studies indicate the involvement of the +III oxidation state of nickel in form of Ni^III^‐chloride,^[9]^ Ni^III^‐oxide/hydroxide,^[10]^ and Ni^III^‐bicarbonate.^[11]^ The formation of a high valent Ni^III^‐oxyl radical species via reaction of *m*CPBA (*m*‐chloroperbenzoic acid) with a Ni^II^ complex bearing a macrocyclic dianionic bisamidate ligand has been reported for C−H oxidation.^[12]^ Hence, accessing oxidation states beyond Ni^III^ may be considered challenging.^[13]^ We thus envisioned that using redox‐active ligands, analogous to metalloenzymes in nature, could allow us to generate Ni complexes bearing more oxidative equivalents than the +III oxidation state permits. Pairing a Ni^III^ species with one‐ and two‐electron oxidized ligands reflects oxidative equivalents of formal Ni^IV^ and formal Ni^V^ species, respectively.

We therefore sought to explore the oxidation chemistry of a Ni^II^ complex bearing the dianionic, redox active, Jacobsen salen ligand, Ni^II^(salen) (salen=*N*,*N′*‐bis(3,5‐di‐*tert*‐butyl‐salicylidene)‐1,2‐cyclohexane‐(1*R*,2*R*)‐diamine), **[1^II^‐L]** or simply **1** (Figure 1c). While the electrochemical oxidation of square planar Ni^II^(salen) to Ni^III^(salen) has been reported,[^14^, ^15^] it was later found that the center of oxidation could be shifted from the metal to the ligand by minimal changes in the experimental conditions. Specifically, in non‐coordinating (CH_2_Cl_2_) and weakly coordinating solvents (CH_3_CN), ligand oxidation is preferred, whereas, with coordinating solvents (DMF, Pyridine, DMSO), the oxidation takes place at the metal center.^[16]^ The behaviour of **1** was investigated by spectroelectrochemical studies in CH_2_Cl_2_, demonstrating the formation of a Ni^III^ phenolate species, **[1^III^‐L]**. The EPR study of this electrochemically generated species showed a temperature‐dependent equilibrium between **[1^III^‐L]** and a Ni^II^ phenoxyl radical species, **[1^II^‐L**⋅**]**.^[17]^ **[1^II^‐L**⋅**]** was later isolated and characterized crystallographically. EPR analysis of **[1^II^‐L**⋅**]** in CH_2_Cl_2_ showed an isotropic signal with g≈2.0, typical for a Ni^II^ phenoxyl radical species at all temperatures.^[18]^ However, these changes were ascribable to the use of a different oxidative procedure. Interestingly the formation of a Ni^II^ bisphenoxyl diradical species, **[1^II^‐L**⋅⋅**]**, was reported by treating **1** with 2 eq. aminium radical cation [N(C_6_H_3_Br_2_)_3_⋅]^+^ in CH_2_Cl_2_ (Figure 1c).^[22]^ Since the precursor (**[1^II^‐L]**, or simply **1** as in Figure 1c) is a neutral complex, the charge of **[1^II^‐L**⋅**]** and **[1^II^‐L**⋅⋅**]** is +1 and +2, respectively. Please note that in our compact notation, charges are omitted for brevity.

Herein, we used *m*CPBA to oxidize **1** at −40 °C. Electrochemical studies, corroborated with UV/Vis, EPR, and resonance Raman spectroscopy, led us to propose the formation of an intriguing Ni^III^ bisphenoxyl diradical species, **[1^III^‐L**⋅⋅**]**. A detailed Intrinsic Bonding Orbital (IBO) analysis based on DFT calculations supports the picture of two‐electron oxidation of the ligand and one‐electron oxidation of the nickel center. **[1^III^‐L**⋅⋅**]** was investigated towards hydrogen atom abstraction (HAA) and oxygen atom transfer (OAT) reactivity.

## Results and Discussion

**Figure 2:**
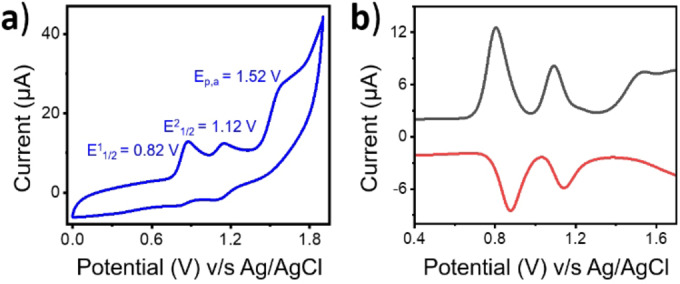
a) Cyclic voltammogram at a scan rate of 100 mV s^−1^ and b) DPV of **1** in CH_3_CN at room temperature. Potentials are referenced vs. Ag/AgCl*. Conditions used*: 0.4 mM **1** in CH_3_CN and 100 mM of tetra‐n‐butylammonium perchlorate (TBAClO_4_) as the supporting electrolyte.

The electrochemical properties of **1** were investigated in CH_3_CN at different scan rates (Figures 2a and S1). **1** exhibits two reversible redox waves at *E* ^1^_1/2_=0.82 and *E* ^2^_1/2_=1.12 V vs. Ag/AgCl. Shimazaki and co‐workers report that the two redox reversible waves can be assigned to Ni⋅^,II/II^ and Ni⋅⋅^,II/^⋅^,II^ redox events, respectively.^[22]^ However, in addition to two reversible redox waves, we also observed an irreversible oxidation wave at *E*_p,a_=1.52 V vs. Ag/AgCl in the cyclic voltammogram and differential pulse voltammetry (DPV) studies (Figure 2b). Since both the phenolate rings and the metal center in **1** are susceptible to oxidation, this preliminary information points towards the formation of a Ni^III^‐diradical species.

As indicated in previous studies, the use of Ce^IV^ or AgSbF_6_, and [N(C_6_H_3_Br_2_)_3_⋅]^+^ (magic blue), as one‐electron oxidants with **1**, made the one and two‐electron oxidized complexes accessible, respectively.[^17^, ^22^] However, the additional redox event we observed in the cyclic voltammetry (Figure 2a) steered us towards employing a different approach. Specifically, we followed the oxidation of **1** by *m*CPBA in CH_3_CN and CH_2_Cl_2_ at −40 °C via UV/Vis absorption spectroscopy.

**Figure 3:**
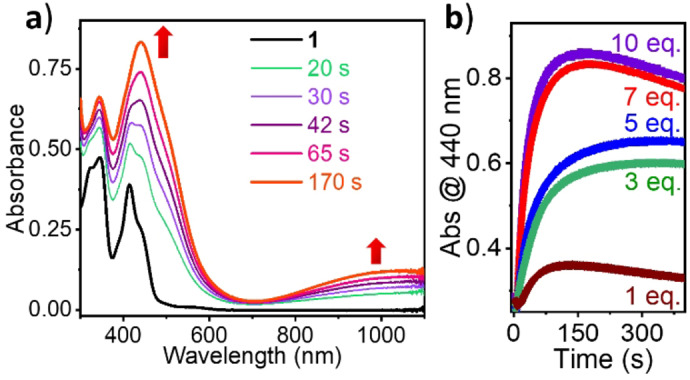
a) Absorption spectra of 0.05 mM **1** on the addition of 7 eq. *m*CPBA turning to 440 nm species in CH_3_CN at −40 °C; 0 s (black, **1**), 20 s (green), 30 s (purple), 42 s (violet), 65 s (pink) and 170 s (orange). b) Absorption changes followed at 440 nm upon adding various equivalents of *m*CPBA to **1** (0.05 mM in CH_3_CN) as indicated in the legend.

The initial Ni^II^ complex exhibits a band at 416 nm along with a shoulder at 460 nm in CH_3_CN, typical for Ni^II^ salen complexes (Figure 3a). The addition of *m*CPBA to **1** resulted in an increase in the unique absorption bands at 440 nm to form an orange‐colored species (Figure 3a). In addition to the 440 nm band, a low energy Near IR transition at 1000 nm suggests the presence of a phenoxyl radical in the system in accordance with the literature.[^17^, ^22^] The yield of this orange‐colored species was observed to be dependent on the concentration of *m*CPBA, saturating at 10 eq. (Figures 3b and S3) with ϵ_440nm_≈18 000 M^−1^ cm^−1^. However, for the present study, 7 eq. *m*CPBA was chosen to be the optimized condition (Figure S4). The 440 nm species is metastable at −40 °C having a *t*_1/2_ value of 50 min (Figure S5), which decays with increasing temperatures. It is also notable that an intense band with a molar absorptivity 18 000 M^−1^ cm^−1^ is different from the reported molar absorptivity values of the mono‐ and bis‐phenoxyl radical species, i.e., **[1^II^‐L**⋅**]** and **[1^II^‐L**⋅⋅**]**.[^17^, ^22^] Based on the electrochemical analysis (see above) and the high molar absorptivity, we hypothesize that this absorption band originates from a species containing more than two oxidizing equivalents. The same reaction was performed in CH_2_Cl_2_ and observations were indistinguishable from those of CH_3_CN (Figures S6 and S7). However, in DMF, the addition of *m*CPBA did not show any change in the spectrum, indicating the unreactive nature of **1** in DMF (Figure S8).

To estimate the number of oxidizing equivalents present in the 440 nm absorbing species, we titrated it with ferrocene (Fc) and acetyl ferrocene (AcFc). Our electrochemical analysis of **1** in CH_3_CN revealed three oxidation events occurring at a potential higher than that of Fc (0.36 V vs. Ag/AgCl). In principle, the ferrocenium equivalents generated upon the reaction of the 440 nm species with ferrocenes allow us to estimate the number of oxidizing equivalents incorporated. When 3 eq. Fc were added to the 440 nm absorbing species, 2.4 eq. ferrocenium ions were observed (Figures S9 and S10). The addition of similar equivalents of AcFc to 440 nm species resulted in 2.4 eq. acetyl ferrocenium (Figure S11). These observations, along with the concomitant regeneration of ≈80 % concentration of the starting Ni^II^ complex **1**, suggest that **1** has gained three oxidizing equivalents upon reaction with 7 eq. *m*CPBA *(*see below*)*.

In both cases, the number of ferrocenium equivalents corresponds to three oxidative equivalents per complex, in line with an assignment of **[1^III^‐L**⋅⋅**]**, **[1^IV^‐L**⋅**]**, or **[1^V^‐L]**. None of the available studies on Ni as the central metal ion have outlined the appearance of a Ni^V^ species thus far. In the present study, the redox‐activity of the salen ligand makes a Ni^V^ assignment even less likely. Hence, assigning the 440 nm absorbing species as **[1^V^‐L]** can be excluded. Formation of **[1^II^‐L**⋅⋅**]**, via oxidation with 2 eq. magic blue, revealed that the salen ligand is readily oxidized.^[22]^ As a result, the assignment of the 440 nm species as **[1^IV^‐L**⋅**]** can also be excluded. Based on the above observations, we propose that two of the oxidizing equivalents reside on the phenolate rings of the ligand. Since the formed 440 nm absorption band with high molar absorptivity is unique, we believe that the Ni^II^ center is oxidized to Ni^III^, leading to a species possessing a total of three oxidizing equivalents.

**Figure 4:**
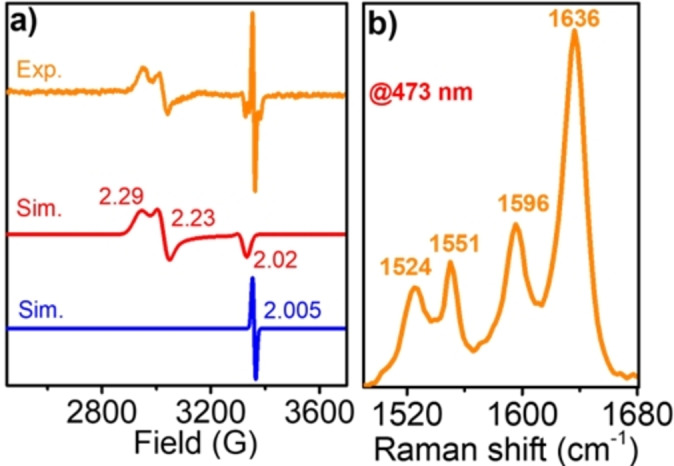
a) X‐band EPR of **[1^III^‐L**⋅⋅**]** at −150 °C. *Condition to generate 440* *nm species*: 2 mM **1**+7 eq. *m*CPBA in CH_2_Cl_2_ at −40 °C. Modulation amplitude 1.98 G; Modulation frequency 100 KHz, and Attenuation 20 dB. *Simulation details*: Red: g_*x*_=2.29, g_*y*_=2.23 and g_*x*_=2.02, g_avg_=2.19 and Blue: g_iso_=2.005. b) Resonance Raman spectrum of **[1^III^‐L**⋅⋅**]** at λ_exc_ 473 nm in CH_2_Cl_2_, at −80 °C.

To further support our assignment, X‐band EPR spectra were recorded at −150 °C for the 440 nm species in CH_2_Cl_2_ and CH_3_CN (Figures 4a and S12). A rhombic signal with g=2.29, 2.23, 2.02 and another isotropic signal with g_iso_=2.005 were found. The previously reported [Ni(3,5‐Cl_2_saloph)]^+^ (3,5‐Cl_2_saloph=bis(3,5‐dichlorosalicylaldehyde)‐o‐phenylenediimine) exhibits a rhombic signal with g_*x*_=2.238, g_*y*_=2.204, and g_*z*_=2.023 in DMF, but g_*x*_ *=*2.234, g_*y*_=2.204, and g_*z*_=2.020 in DMSO, consistent with a Ni^III^ species.^[23]^ A study on **1** reported g_avg_=2.17 for **[1^III^‐L]**, of an S=1/2 species at temperatures below −120 °C in CH_2_Cl_2_, and at all temperatures in DMF.^[17]^ However, reported EPR spectra of **[1^II^‐L**⋅**]** in CH_2_Cl_2_ at temperatures above −100 °C show an isotropic signal at g_iso_=2.04.^[17]^ After isolation of the species, the same isotropic signal was found.^[18]^ Furthermore, **[1^II^‐L**⋅⋅**]** is reported to be EPR silent due to two antiferromagnetically coupled electrons.^[22]^ The existing literature hence includes both a metal‐centered oxidized species, **[1^III^‐L]**, and the ligand oxidized species, **[1^II^‐L**⋅**]**, which can be interconverted by minor changes in either the temperature or solvent. Our EPR spectrum of the three electron oxidized species (Figure 4a) shows an isotropic signal at g_iso_=2.005, which is expected for **[1^II^‐L**⋅**]** at the first instance. However, the different UV/Vis absorption bands, with ≈3 eq. ferroceniums and the concomitant regeneration of 80 % **1** suggest the formation of a three electron oxidized species. Therefore, the isotropic signal in the present case can be assigned to **[1^III^‐L**⋅⋅**]**, where three unpaired electrons are antiferromagnetically coupled, resulting in an effective S=1/2 electron localized on one of the phenolate rings of the ligand. In addition, the appearance of a rhombic signal bearing a g_avg_=2.19, which is slightly different from the reported **[1^III^‐L]**, can also be assigned to a **[1^III^‐L**⋅⋅**]** species, with the effective S=1/2 electron residing on the metal center. Time‐resolved EPR spectroscopy clearly shows the formation and decay of these species in the same timescale as observed by UV/Vis spectroscopy (Figure S12b). Hence, EPR suggests the coexistence of a species with an unpaired electron localized on the Ni and a species with a ligand localized radical. The presence of a ferromagnetically coupled system can be excluded since it would give an S=3/2 species with a higher g value than what we observed in the current case. DFT studies further supported this analysis (see below), where configurations with Ni‐centered and ligand‐centered effective spin were observed to be close in energy.

Resonance Raman (rR) spectroscopy was employed to probe the nature of the 440 nm electronic transition and the existence of a diradical species. The rR spectrum of **[1^III^‐L**⋅⋅**]** at λ_exc_ 473 nm revealed several resonantly enhanced bands (1640–800 cm^−1^) that originate from the salen ligand (Figures 4b and S13).^[24]^ Notably, the bands at 1524 & 1551 cm^−1^ and 1596 & 1636 cm^−1^ fall in the range of C−O and C=C stretches of the ligand, respectively. These are indicative of the existence of a phenoxyl radical. It was reported that oxidation of the ligand results in a decrease of the Raman shift of the C=C stretch from 1627 cm^−1^ to 1579 cm^−1^, upon oxidation of **[1^II^‐L]** to **[1^II^‐L**⋅⋅**]**.[^17^, ^22^] In the current case, the band at 1596 cm^−1^ was found to match this scenario. However, the band at 1636 cm^−1^ is blue‐shifted compared to the parent Ni^II^ complex **1** (1622 cm^−1^). The two sets of signals were tentatively assigned to the two species (*species 1*: effective spin localized on ligand and *species 2*: effective spin localized on the metal) that exist in equilibrium, as suggested by our EPR and DFT studies (see below). Hence, rR indicates the existence of nickel‐bound phenoxyl radicals in **[1^III^‐L**⋅⋅**]**. Moreover, the selective enhancement of the ligand vibrational modes suggests that the 440 nm band is due to salen ligand‐to‐metal charge transfer.

We computationally investigated the three‐fold oxidized Ni species via DFT calculations combined with an Intrinsic Bonding Orbital (IBO)^[25]^ analysis. The structural parameters were optimized at the M06‐L^[26]^/def2‐SVP^[27]^ level of theory, and the electronic structure was subsequently analyzed with the larger def2‐TZVPP basis set. Assuming no axial ligands, our triply oxidized species has an intrinsic *d* ^*8*^ Ni^II^ configuration implying a three‐fold ligand oxidation (Figure S14 for IBO analysis). This represents an apparent inconsistency with the measured EPR and rR spectra of the triply oxidized species, which indicated metal‐based oxidation and an overall S=1/2 spin state.

**Figure 5:**
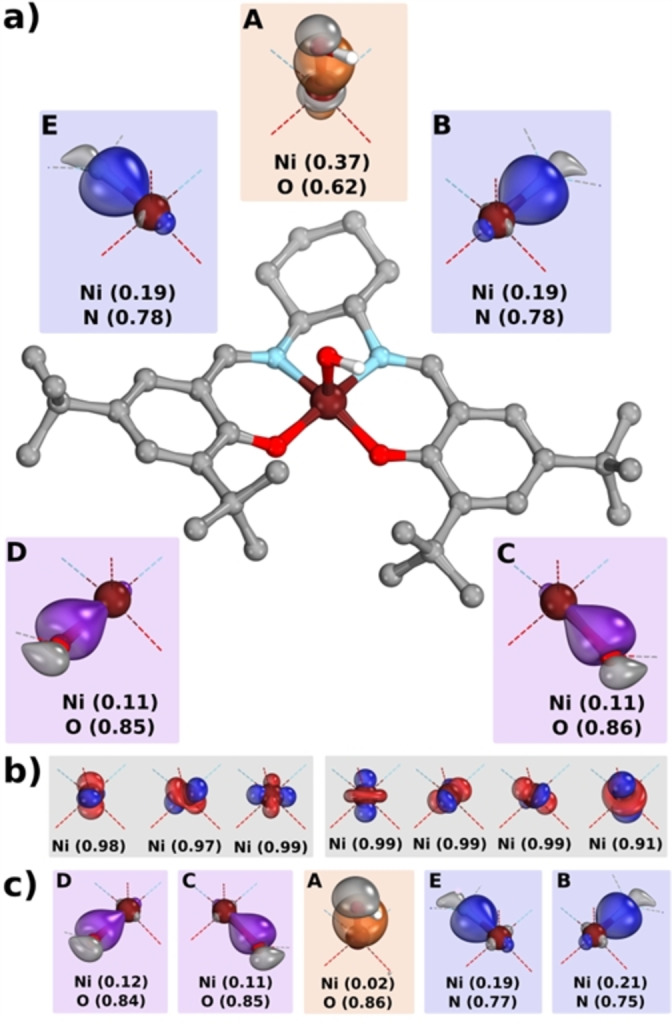
IBO analysis of **[1^III^‐L**⋅⋅**]** bearing a hydroxide axial ligand in its lowest energy state (low spin). The metal‐ligand σ‐bonding orbitals are shown in the α‐space (a), and the β‐space (c). The intrinsic d^7^ configuration (b), comprised of 3×α(δ‐IBO) (left) and 4×β(δ‐IBO) (right). Calculated with M06‐L/def2‐TZVPP/cPCM//M06‐L/def2‐SVP/PCM, isosurfaces rendered in IboView v2021^[29]^ to enclose 80 % of each orbital's electron density. Hydrogen atoms are omitted for clarity.

**Figure 6:**
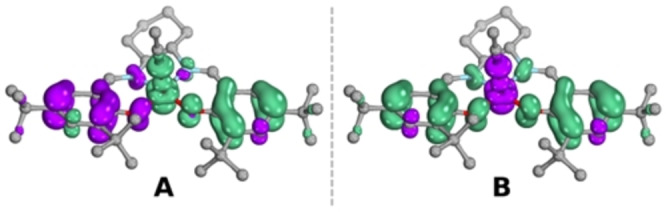
Spin density plots of the energetically near‐degenerate solutions of five‐coordinate **[1^III^‐L**⋅⋅**]** bearing an acetonitrile axial ligand: **LS_A_** (A) and **LS_B_** (B), calculated with M06‐L/def2‐TZVPP/cPCM//M06‐L/def2‐SVP/PCM in ORCA 5.0.1^[30, 31]^//Gaussian 16^[32]^ Positive spin density is depicted in green and negative spin density in purple (isosurface 0.004). Hydrogen atoms are omitted for clarity.

In order to address this, we also considered five‐ and six‐coordinate structures featuring hydroxide and acetonitrile ligands, which may be bound under the reaction conditions. Given the large positive charge of the triply oxidized complex, the anionic hydroxide is a likely candidate for the axial ligand, which we also probe through HAA reactivity, see below. With a hydroxide bound, we found an intrinsic *d* ^*7*^ Ni^III^ configuration (Figure 5), where two‐electron oxidation of the ligand occurred together with one‐electron oxidation of the metal center. The low spin (LS) state was found to be lower in energy than the intermediate spin (IS) by 4.8 kcal mol^−1^ (see Table S2 for the energetics of all species). Therefore, the calculated electronic structure is consistent with the S=1/2 species measured spectroscopically, which we assign as **[1^III^‐L**⋅⋅**]**. The same conclusion for ligand and metal oxidation can be drawn when considering a neutral axial ligand, as in CH_3_CN (Figures S16 and S17). Two energetically near‐degenerate electronic solutions were found at the optimized LS geometry (Figure 6, for corresponding orbitals see Figures S22 and S23),^[28]^ both of which still correspond to intrinsic *d* ^*7*^ Ni^III^ configurations and differ only in spin coupling patterns (consistent with the EPR and rR data). Hexa‐coordinated complexes featuring two axial ligands (hydroxide or CH_3_CN) equally show electronic structures consistent with mixed metal and ligand oxidation (Figures S18–20)_._ ^[29]^ We note that computationally predicting a spin ground state can be challenging and, in several instances, can exhibit high sensitivity to the chosen functional (see section 4.2.1 in the Supporting Information for more details). Nevertheless, these calculations are informative as they allow the implications of oxidation on the electronic structure to be probed. Specifically, these results show that no axial ligand binding in the triply oxidized species is inconsistent with the spectroscopic observations, implying that an axial ligand must be present.

**[1^III^‐L**⋅⋅**]** was tested for its efficiency towards HAA and OAT with different classes of substrates (Table 1). In the case of 2,4,6‐tri‐tert‐butylphenol (2,4,6‐TTBP), pseudo‐first‐order rate constants were obtained (Figure S24). The reaction resulted in an exponential decay in the absorbance at 440 nm of **[1^III^‐L**⋅⋅**]** with an accompanied increase in the absorption bands at 385 nm, 403 nm, and 626 nm (Figure S26). The new absorption bands were found to be characteristic of the 2,4,6‐TTBP radical species (2,4,6‐TTBP^**•**+^).^[34]^ The reaction of *m*CPBA alone with 2,4,6‐TTBP did not proceed under the same conditions (Figure S27). The obtained value of *k*_obs_ varied proportionally with substrate concentration, leading to a second‐order rate constant (*k*_2_/M^−1^s^−1^) of 30 M^−1^s^−1^ (Figure S25). **[1^III^‐L**⋅⋅**]** is also capable of oxidizing thioanisole (Figures S28–S30) and PPh_3_ (Table 1, Figures S31–S33), substrates typically used to probe OAT reactivity. The rate of PPh_3_ oxidation is 215‐fold higher than that of thioanisole. Under similar conditions, *m*CPBA alone gave only sulfoxide and Ph_3_P=O (triphenyl phosphine oxide) with thioanisole and PPh_3,_ respectively, as the oxidized product leading to the background reactivity (Figure S30 and S33). However, the rate of consumption of **[1^III^‐L**⋅⋅**]** (monitored by UV/Vis spectroscopy) scales linearly with substrate concentration, indicating that although excess *m*CPBA is present, **[1^III^‐L**⋅⋅**]** does exhibit some oxidation reactivity with these substrates.

**Table 1:** Phenol O−H bond activation, HAA, and OAT reactivity of **[1^III^‐L**⋅⋅**]** in CH_3_CN at −40 °C.

Class of reaction	Substrates	*k*_2_ [M^−1^ s^−1^]
O−H activation	2,4,6‐TTBP	30
OAT reactivity	Thioanisole	0.88
	PPh_3_	190
C−H activation	Xanthene	0.91

The reaction of **[1^III^‐L**⋅⋅**]** with substrates bearing activated C−H bonds was also examined. The addition of different equivalents of xanthene (100–400 eq.) to the 440 nm species at −40 °C resulted in the decay of its absorbance (Figures S34–S36), again obeying pseudo first‐order kinetics with *k*_2_ of 0.91 M^−1^s^−1^. HPLC analysis showed the formation of xanthone as the oxidized product, which was seen to be absent in the blank reaction (Figure S36). The incorporation of oxygen in the oxidized product of xanthene indicates the presence of a oxygen derived axial ligand, most likely a *hydroxide*, in **[1^III^‐L**⋅⋅**]**. DFT calculations of such a species also provided an electronic structure description that is consistent with the experimental results (see above). To the best of our knowledge, no reactivity studies have been reported for the oxidized Ni salen species (**[1^II^‐L**⋅**]**, **[1^II^‐L**⋅⋅**]** or **[1^III^‐L**⋅⋅**]**), which makes the present study crucial, since it shows that **[1^III^‐L**⋅⋅**]** could be used as the oxidant in reactions with substrates typically used for HAA and OAT reactions.

## Conclusion

This study reports the accessibility of an unprecedented high valent Ni^III^ bisphenoxyl diradical species **[1^III^‐L**⋅⋅**]**. The presence of the dianionic and redox‐active Jacobsen salen ligand enables the Ni^II^ precursor to incorporate three oxidative equivalents by acting as an electron reservoir. Electrochemical and spectroscopic studies (specifically: UV/Vis, EPR, and rR), coupled with an IBO analysis based on DFT calculations, substantiate the formation of a fleeting Ni^III^ complex bearing a diradical salen ligand. **[1^III^‐L**⋅⋅**]** exhibits reactivity with ferrocenes, the trisubstituted phenol 2,4,6‐TTBP, an activated C−H bond (in xanthene), and typical oxo transfer substrates (thioanisole, and PPh_3_). We thus conclude that **[1^III^‐L**⋅⋅**]** has potential as an oxidant that incorporates multiple oxidative equivalents without the need to access a high valent state.

## Conflict of interest

The authors declare no conflict of interest.

1

## Supporting information

As a service to our authors and readers, this journal provides supporting information supplied by the authors. Such materials are peer reviewed and may be re‐organized for online delivery, but are not copy‐edited or typeset. Technical support issues arising from supporting information (other than missing files) should be addressed to the authors.

Supporting Information

## Data Availability

The data that support the findings of this study are available in the Supporting Information of this article.

## References

[1a] N. Ito , S. E. V. Phillips , K. D. S. Yadav , P. F. Knowles , J. Mol. Biol. 1994, 238, 794–814; 8182749 10.1006/jmbi.1994.1335

[1b] N. P. Dunham , F. H. Arnold , ACS Catal. 2020, 10, 12239–12255; 33282461 10.1021/acscatal.0c03606 PMC7710332

[1c] A. Das , C. Hessin , Y. Ren , M. Desage-El Murr , Chem. Soc. Rev. 2020, 49, 8840–8867; 33107878 10.1039/d0cs00914h

[1d] A. C. Ghosh , C. Duboc , M. Gennari , Coord. Chem. Rev. 2021, 428, 213606.

[2a] S. Itoh , M. Taki , S. Fukuzumi , Coord. Chem. Rev. 2000, 198, 3–20;

[2b] W. Kaim , Bioinorganic chemistry—Inorganic Elements in the Chemistry of Life: An Introduction and Guide, 2 ^nd^ ed., Wiley, Hoboken, 2013.

[3a] P. J. Chirik , Inorg. Chem. 2011, 50, 9737–9740; 21894966 10.1021/ic201881k

[3b] W. Kaim , B. Schwederski , Coord. Chem. Rev. 2010, 254, 1580–1588;

[3c] W. Kaim , Eur. J. Inorg. Chem. 2012, 3, 343–348.

[4a] J. H. Dawson , M. Sono , Chem. Rev. 1987, 87, 1255–1276;

[4b] B. Meunier , S. P. de Visser , S. Shaik , Chem. Rev. 2004, 104, 3947–3980; 15352783 10.1021/cr020443g

[4c] X. Huang , J. T. Groves , Chem. Rev. 2018, 118, 2491–2553; 29286645 10.1021/acs.chemrev.7b00373 PMC5855008

[4d] T. H. Yosca , J. Rittle , C. M. Krest , E. L. Onderko , A. Silakov , J. C. Calixto , R. K. Behan , M. T. Green , Science 2013, 342, 825–829; 24233717 10.1126/science.1244373 PMC4299822

[4e] P. W. Gingrich , J. B. Siegel , D. J. Tantillo , J. Chem. Inf. Model. 2022, 62, 1979–1987. 35421306 10.1021/acs.jcim.1c01567

[5] J. A. Halfen , B. A. Jazdzewski , S. Mahapatra , L. M. Berreau , E. C. Wilkinson , L. Que Jr , W. B. Tolman , J. Am. Chem. Soc. 1997, 119, 8217–8227.

[6a] L. Que Jr. , Bull. Jpn. Soc. Coord. Chem. 2013, 62, 30–37; 25678937 PMC4322783

[6b] W. N. Oloo , L. Que , Acc. Chem. Res. 2015, 48, 2612–2621; 26280131 10.1021/acs.accounts.5b00053

[6c] G. Mukherjee , J. K. Satpathy , U. K. Bagha , M. Q. E. Mubarak , C. V. Sastri , S. P. De Visser , ACS Catal. 2021, 11, 9761–9797;

[6d] L. Vicens , M. Costas , Dalton Trans. 2018, 47, 1755–1763; 29344593 10.1039/c7dt03657d

[6e] M. Guo , Y. M. Lee , S. Fukuzumi , W. Nam , Coord. Chem. Rev. 2021, 435, 213807;

[6f] J. E. M. N. Klein , L. Que Jr , Biomimetic High-Valent Mononuclear Non-heme Iron-Oxo Chemistry, in Encyclopaedia of Inorganic and Bioinorganic Chemistry (Ed.: R. A. Scott ), Wiley, Chichester, 2016;

[6g] G. C. Allen , K. D. Warren , Inorg. Chem. 1969, 8, 753–756;

[6h] N. A. Matwiyoff , L. B. Asprey , W. E. Wageman , M. J. Reisfeld , E. Fukushima , Inorg. Chem. 1969, 8, 750–753.

[7] M. Sankaralingam , M. Balamurugan , M. Palaniandavar , P. Vadivelu , C. H. Suresh , Chem. Eur. J. 2014, 20, 11346–11361. 25100547 10.1002/chem.201402391

[8a] S. Z. Tasker , E. A. Standley , T. F. Jamison , Nature 2014, 509, 299–309; 24828188 10.1038/nature13274 PMC4344729

[8b] N. Nebra , Molecules 2020, 25, 1141–1172.32143336

[9] P. Mondal , P. Pirovano , A. Das , E. R. Farquhar , A. R. McDonald , J. Am. Chem. Soc. 2018, 140, 1834–1841. 29293330 10.1021/jacs.7b11953

[10] F. F. Pfaff , F. Heims , S. Kundu , S. Mebs , K. Ray , Chem. Commun. 2012, 48, 3730–3732. 10.1039/c2cc30716b 22398975

[11] P. Pirovano , E. R. Farquhar , M. Swart , A. R. McDonald , J. Am. Chem. Soc. 2016, 138, 14362–14370. 27739688 10.1021/jacs.6b08406 PMC5119486

[12] T. Corona , F. F. Pfaff , F. Acuña-Parés , A. Draksharapu , C. J. Whiteoak , V. Martin-Diaconescu , J. Lloret-Fillol , W. R. Browne , K. Ray , A. Company , Chem. Eur. J. 2015, 21, 15029–15038. 26311073 10.1002/chem.201501841

[13] J. S. Steen , G. Knizia , J. E. M. N. Klein , Angew. Chem. Int. Ed. 2019, 58, 13133–13139; 10.1002/anie.201906658 PMC6771483 31206937

[14] B. De Castro , C. Freire , E. Pereira , J. Chem. Soc. Dalton Trans. 1994, 571–576.

[15] C. Freire , B. De Castro , J. Chem. Soc. Dalton Trans. 1998, 1491–1497.

[16] F. Thomas , Dalton Trans. 2016, 45, 10866–10877. 27077646 10.1039/c6dt00942e

[17] Y. Shimazaki , F. Tani , K. Fukui , Y. Naruta , O. Yamauchi , J. Am. Chem. Soc. 2003, 125, 10512–10513. 12940721 10.1021/ja035806o

[18] T. Storr , E. C. Wasinger , R. C. Pratt , T. D. P. Stack , Angew. Chem. Int. Ed. 2007, 46, 5198–5201; 10.1002/anie.200701194 17546576

[19] K. Ray , T. Weyhermüller , F. Neese , K. Wieghardt , Inorg. Chem. 2005, 44, 5345–5360. 16022533 10.1021/ic0507565

[20] M. Ghosh , T. Weyhermüller , K. Wieghardt , Dalton Trans. 2008, 38, 5149–5151. 10.1039/b813192a 18813366

[21] D. Herebian , E. Bothe , F. Neese , T. Weyhermüller , K. Wieghardt , J. Am. Chem. Soc. 2003, 125, 9116–9128. 15369369 10.1021/ja030123u

[22] T. J. Dunn , M. I. Webb , K. Hazin , P. Verma , E. C. Wasinger , Y. Shimazaki , T. Storr , Dalton Trans. 2013, 42, 3950–3956. 23334388 10.1039/c2dt32632a

[23] B. De Castro , C. Freire , Inorg. Chem. 1990, 29, 5113–5119.

[24a] M. L. McGlashen , D. D. Eads , T. G. Spiro , J. W. Whittaker , J. Phys. Chem. 1995, 99, 4918–4922;

[24b] T. Storr , P. Verma , R. C. Pratt , E. C. Wasinger , Y. Shimazaki , T. D. P. Stack , J. Am. Chem. Soc. 2008, 130, 15448–15459; 18939830 10.1021/ja804339m PMC2663632

[24c] C. R. Johnson , M. Ludwig , S. A. Asher , J. Am. Chem. Soc. 1986, 108, 905–912;

[24d] A. Sokolowski , J. Müller , T. Weyhermüller , R. Schnepf , P. Hildebrandt , K. Hildenbrand , E. Bothe , K. Wieghardt , J. Am. Chem. Soc. 1997, 119, 8889–8900.

[25] G. Knizia , J. Chem. Theory Comput. 2013, 9, 4834–4843. 26583402 10.1021/ct400687b

[26] Y. Zhao , D. G. Truhlar , Theor. Chem. Acc. 2008, 120, 215–241.

[27] F. Weigend , R. Ahlrichs , Phys. Chem. Chem. Phys. 2005, 7, 3297–3305. 16240044 10.1039/b508541a

[28] F. Neese , J. Phys. Chem. Solids 2004, 65, 781–785.

[29] For the bishydroxide complex **[1^III^-L**⋅⋅**−(OH)_2_]** we do note some functional dependence (see Supporting Information for further details).

[30] F. Neese , WIREs Comput. Mol. Sci. 2012, 2, 73–78.

[31] F. Neese , WIREs Comput. Mol. Sci. 2018, 8, e1327.

[32] Gaussian16, revision B.01 M. J. Frisch, G. W. Trucks, H. B. Schlegel, G. E. Scuseria, M. A. Robb, J. R. Cheeseman, G. Scalmani, V. Barone, G. A. Petersson, H. Nakatsuji, et al., (for a full list of authors, please refer to the Supporting Information) Gaussian, Inc: Wallingford, CT, **2016**.

[33] F. G. Bordwell , Z. Xian-Man , J. Phys. Org. Chem. 1995, 8, 529–535.

[34] T. Wu , S. N. MacMillan , K. Rajabimoghadam , M. A. Siegler , K. M. Lancaster , I. Garcia-Bosch , J. Am. Chem. Soc. 2020, 142, 12265–12276. 32531159 10.1021/jacs.0c03867 PMC8300862

