# Supplementary material for: Formation and Reactivity of a Fleeting NiIII Bisphenoxyl Diradical Species
Source: Angew Chem Int Ed Engl. 2022 Sep 2;61(41):e202211345. doi: 10.1002/anie.202211345 (PMC9826141; doi:10.1002/anie.202211345)
Supplement: Supplementary file 1 — Supporting Information [file ANIE-61-0-s001.pdf]

Angewandte Chemie
Eine Zeitschrift der Gesellschaft Deutscher Chemiker

Supporting Information

Formation and Reactivity of a Fleeting Ni^{III} Bisphenoxyl Diradical Species

A. Awasthi, I. F. Leach, S. Engbers, R. Kumar, R. Eerlapally, S. Gupta, J. E. M. N. Klein, A. Draksharapu**

Supporting Information

1. Experimental Section

1.1 Materials and Methods

All chemicals and reagents used in the present study that are commercially available were used as received. The ligand, (R,R)-(-)-N,N'-Bis(3,5-di-tert-butylsalicylidene)-1,2-cyclohexanediamine was purchased from Tokyo Chemicals Ltd. (TCI). $NiCl_2$, ferrocene, acetyl ferrocene, *m*CPBA were acquired from Sigma Aldrich and Sisco Research Laboratories (SRL). HPLC grade dry CH_3CN, CH_2Cl_2, and DMF from Thomas Baker were used in the spectroscopic studies.

UV-Vis absorption spectroscopic studies were performed by Agilent 8453 diode-array spectrophotometer to carry out kinetics experiments spectrophotometrically in 1 cm quartz cells (λ = 190 – 1100 nm range). A low temperature of -40 °C and -80 ºC were maintained with a cryostat from Unisoku Scientific Instrument. X-band EPR spectra were recorded at 120 K using a Bruker EMX 1444 spectrometer with a temperature controller. Simulations of the EPR spectra were performed with the WINEPR SimFonia program.

Electrochemical analyses were done at room temperature in CH_3CN through cyclic voltammetry experiments using the CH instrument, Electrochemical Analyzer M-600B series. A three-electrode system, where a glassy carbon (CHI 104 Glassy Carbon Disk Working Electrode) as a working electrode, Pt wire as the counter, and an aqueous Ag/AgCl electrode was used as the reference electrode. The solutions used were ~0.4 mM **1** and 100 mM of tetra-n-butylammonium perchlorate ($TBAClO_4$) as the supporting electrolyte.

Raman spectra were obtained using a λ_{exc} of 473 nm from Cobolt Lasers (75 mW at source). Spectra were recorded using a 180° backscattering arrangement. Raman scattering was collected by a plano-convex lens (2.5 cm diameter, f = 7.5 cm). The collimated Raman scattering was then passed through a long pass edge filter from Semrock and focused by a second plano-convex lens (2.5 cm diameter, f = 7.5 cm) into a Shamrock300i spectrograph from Andor Technology with a 1200 L/mm grating blazed at

500 nm, acquired with an iDus-430-BV CCD camera from Andor Technology. The spectral slit width was set to 50 μm. UV-Vis absorption spectra were obtained simultaneous sly using a fiber coupled deuterium/halogen light source and an Avantes-EVO mini spectrometer. Sample cooling was performed using a CoolSpeK USP-203-B Unisoku cryostat.

The reaction mixture was injected (25 µL) in HPLC system (Agilent 1260 infinity machine model) attached with a ZORBAX SB-C-18 reverse phase column (150 × 4.6 mm, 5 µm) and DAD detector. Individual purity of the sample was analyzed prior to reactivity studies using HPLC experiments with the methods described below: A gradient mixture of water and acetonitrile possessing 0.1% trifluoroacetic acid (pre-degassed by applying vacuum and ultrasonication) was used as the mobile phase. Method: 0 min to 20 min, 0.5 mL per min flow rate. For the oxidation reaction of triphenylphosphine, condition A was used and for oxidation of thioanisole and xanthene, condition B was used.

Condition A:

Time (minutes)	Acetonitrile (0.1% trifluoroacetic acid)	Water (0.1% trifluoroacetic acid)
0-8	60%	40%
8.10	90%	10%
12.00	90%	10%
12.10	80%	20%
15.00	70%	30%
20.00	60%	40%

Condition B:

Time (minutes)	Acetonitrile (0.1% trifluoroacetic acid)	Water (0.1% trifluoroacetic acid)
0-8	60%	40%
8.10	90%	10%
12.00	90%	10%
12.10	90%	10%
15.00	60%	40%
20.00	60%	40%

1.2 Synthesis of Complex 1

1 was obtained by mixing $NiCl_2$ (0.55 mmol) with the ethanolic solution of the ligand (0.55 mmol) at 75 °C. The addition of Et_3N (1.1 mmol) to the above turbid mixture turns the colour from yellow to mustard brown. The reaction was left for stirring for 4 h, following which it was brought to room temperature, and the solvent was evaporated under reduced pressure. Long red needle-like crystals of **1** were obtained in 4-5 days in N, N-dimethylformamide (DMF), and the X-ray crystallographic parameters were found to match those in the literature.[1]

1.3 Reactivity conditions in UV-Vis

To 0.05 mM **1**, 7 eq. *m*CPBA was added to generate 440 nm species at -40 °C in CH_3CN. To the maximum absorbance of 440 nm species, different eq. substrates are added, which caused the decay in its absorbance.

1.4 Catalytic reaction conditions for HPLC analysis

T*o* a solution of **1** (concentration mentioned in the captions), 7 eq. *m*CPBA was added at -40 °C in CH_3CN to generate 440 nm species. The generation is followed by the addition of 10 eq. substrate to it. The reaction mixture was then left to react completely as per the substrate's reaction profile monitored through UV-Vis absorption spectroscopy (Figures S27, S30 and S33). The samples were then stored in Liq. N_2 to avoid further reaction until the HPLC data was recorded. Under the similiar conditions the blank reaction of 10 eq. substrate with 7 eq. *m*CPBA without **1,** was done at -40 °C in CH_3CN.

UV-Vis absorption experiments

The majority of the existing reports on **1** have been carried out using CH_2Cl_2 or DMF as the solvents. For the present study, we used CH_3CN and CH_2Cl_2 as our choice of solvents. However, due to the lesser solubility of **1** in CH_3CN, we performed a series of experiments to evaluate its concentration. $\varepsilon_{416\ nm}$ of **1** in DMF is found to be 7000 M^{-1} cm^{-1}. Therefore, 2 mM of **1** was prepared in DMF, from which it was diluted to 1:19 DMF:CH_3CN ratio. The $\varepsilon_{416\ nm}$ of the same was recorded to be 6800 $M^{-1}cm^{-1}$ which is considerably close to the reported 7000 M^{-1} cm^{-1} epsilon at 416 nm in DMF (Figure S2). Since such dilution experiments yielded consistent results, we estimated **1** to have $\varepsilon_{416\ nm}$ = 7000 M^{-1} cm^{-1} in CH_3CN. The established $\varepsilon_{416\ nm}$ = 7000 M^{-1} cm^{-1} helped us in figuring the exact concentration of **1** in CH_3CN for all our experiments.

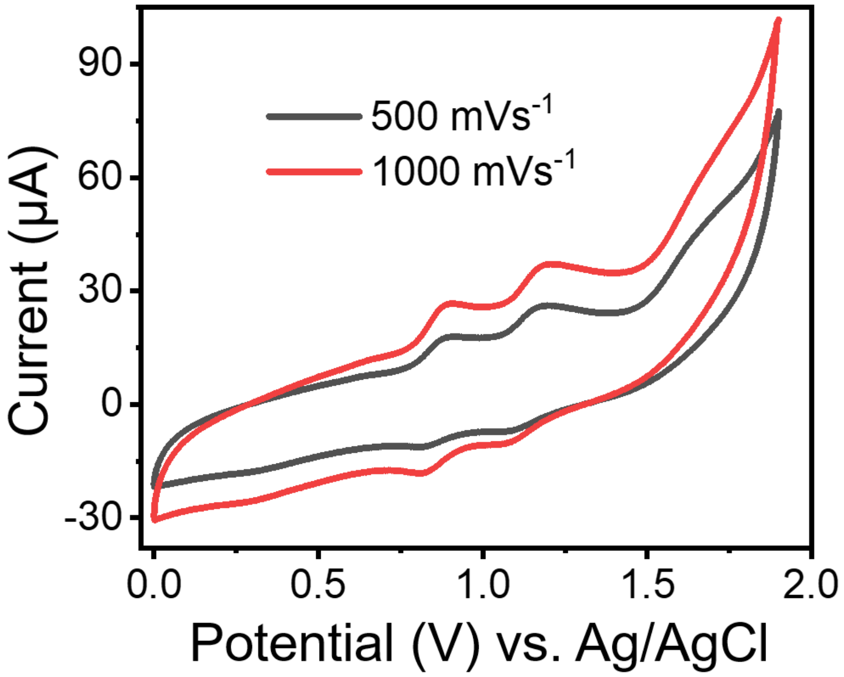

Figure S1. Cyclic voltammograms of **1** in CH_3CN at 500 mV/s (black) and 1000 mV/s (red) scan rates at room temperature.

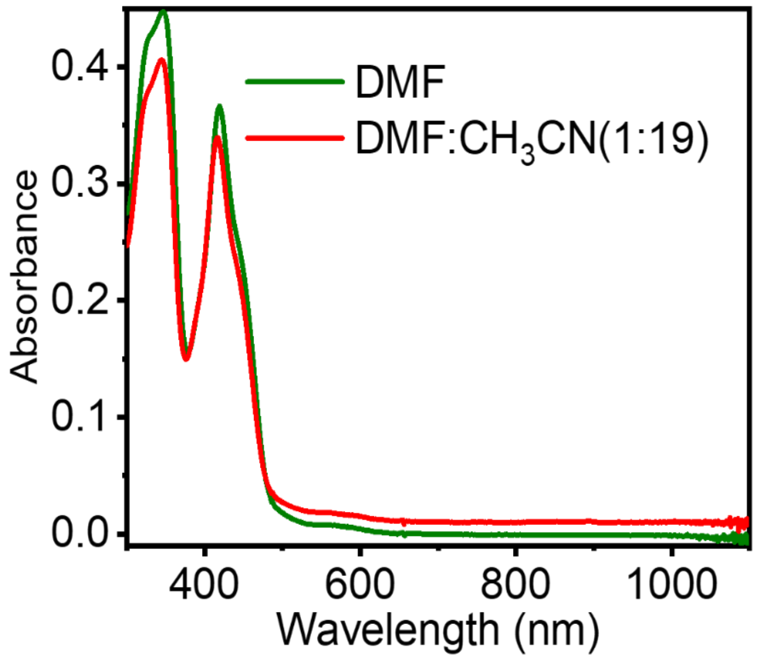

Figure S2. UV-Vis absorption spectra of 0.05 mM **1** in DMF and DMF:CH_3CN (1:19) at room temperature.

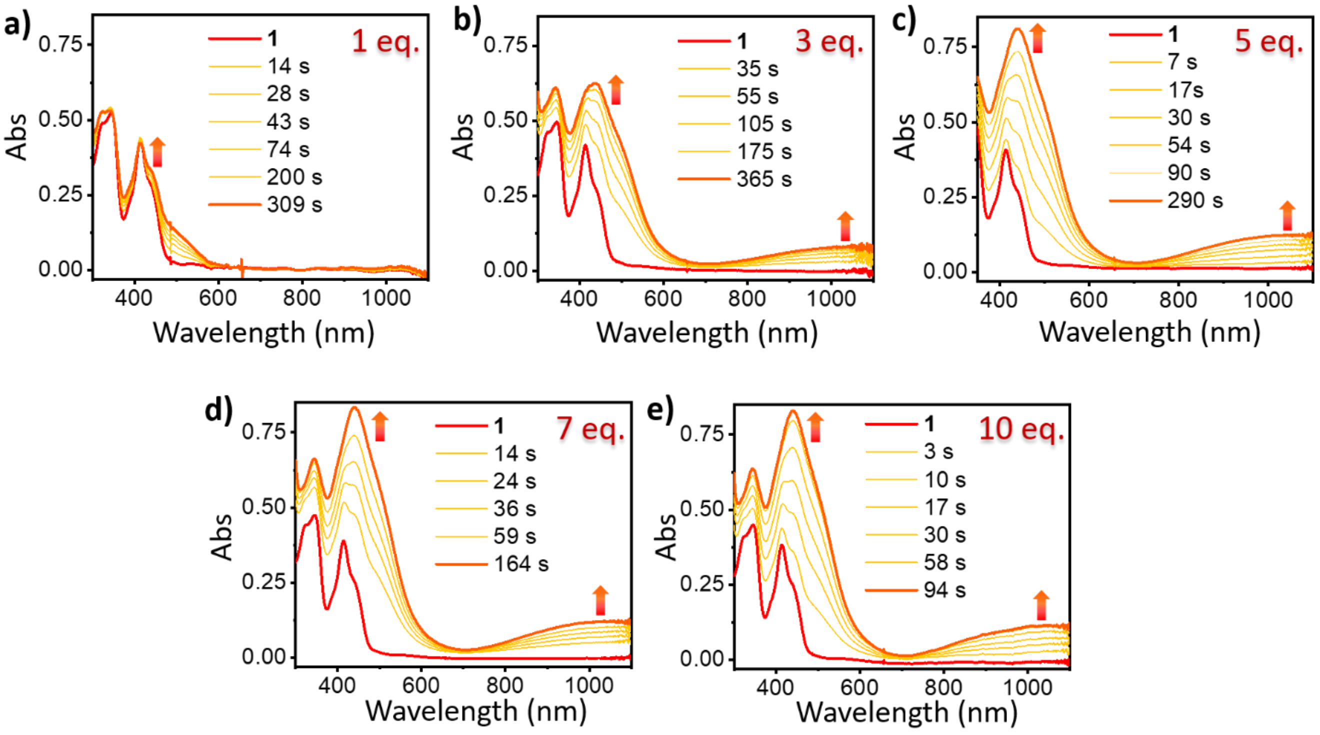

Figure S3. UV-Vis absorption spectra depicting the formation of 440 nm species with different eq. *m*CPBA. *Conditions used:* a) 1 eq., b) 3 eq., c) 5 eq., d) 7 eq., and e) 10 eq. *m*CPBA were added to 0.05 mM **1** in CH_3CN at -40 °C.

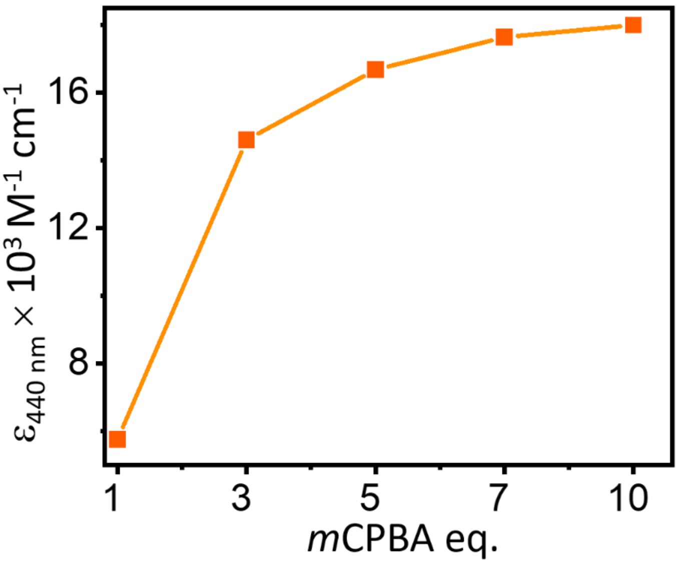

Figure S4. The plot of $\varepsilon_{440\ nm}$ against various equivalents of *m*CPBA.

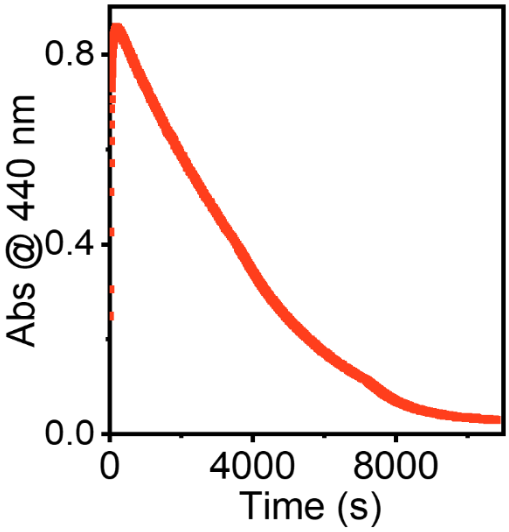

Figure S5. Plot depicting the self-decay of 440 nm species formed from the addition of 7 eq. *m*CPBA to 0.05 mM **1** at -40 °C in CH_3CN. The estimated $t_{1/2}$ is 50 min.

UV-Vis absorption study in CH_2Cl_2 and DMF

When CH_2Cl_2 is used as a solvent, the reaction of **1** with 7 eq. *m*CPBA generated a band at 440 nm with an $\varepsilon_{440\ nm}$ ~ 16,000 M^{-1} cm^{-1} (Figure S8), which is indistinguishable from that of CH_3CN (Figure S3d). The 440 nm species can be assigned as **[1^{III}-L$^{\bullet\bullet}$]** as in the case of CH_3CN. However, in DMF addition of 7 eq. *m*CPBA did not show any change in the spectra stating the unreactive nature of **1** in DMF (Figure S10).

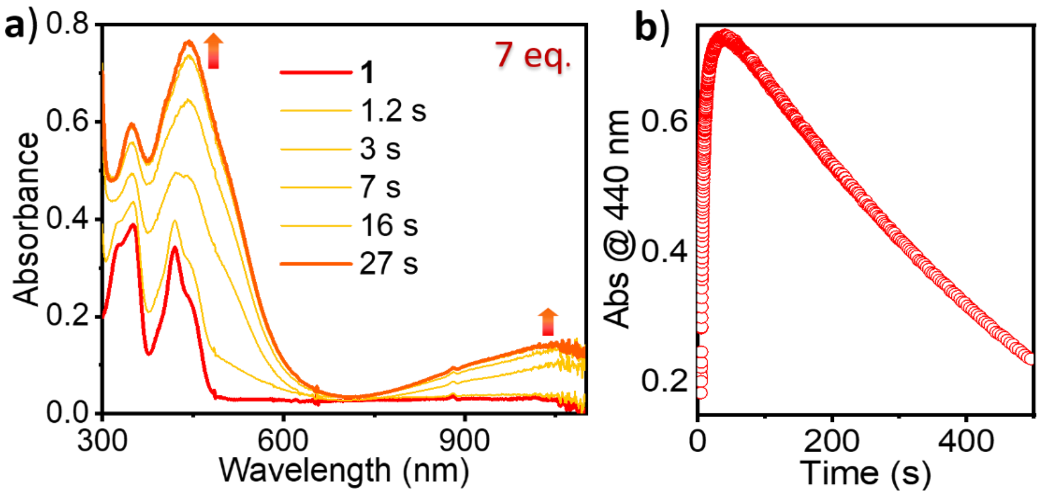

Figure S6. a) Absorption spectra of 0.05 mM **1** upon adding 7 eq. *m*CPBA turning to 440 nm species in CH_2Cl_2 at -40 °C. b) The corresponding absorption changes followed at 440 nm upon the addition of 7 eq. *m*CPBA.

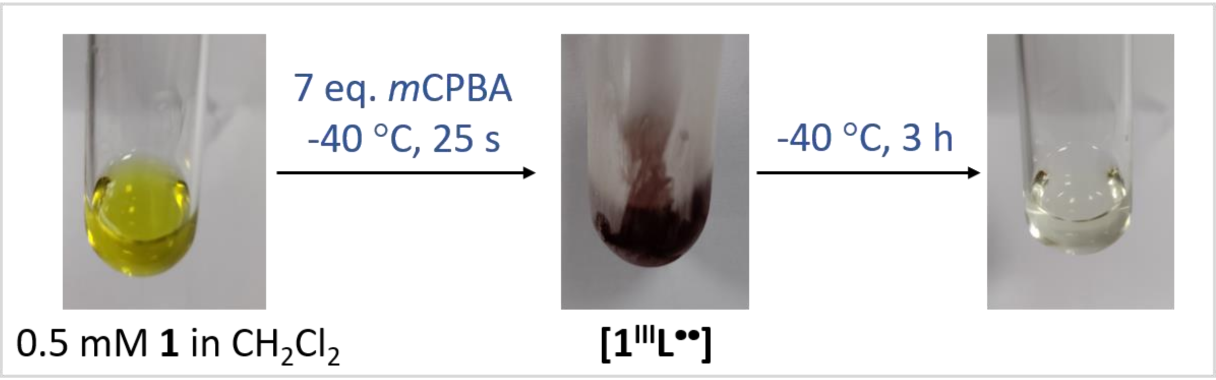

Figure S7. Picture representation for the formation of 440 nm species at -40 °C followed by its decay in CH_2Cl_2.

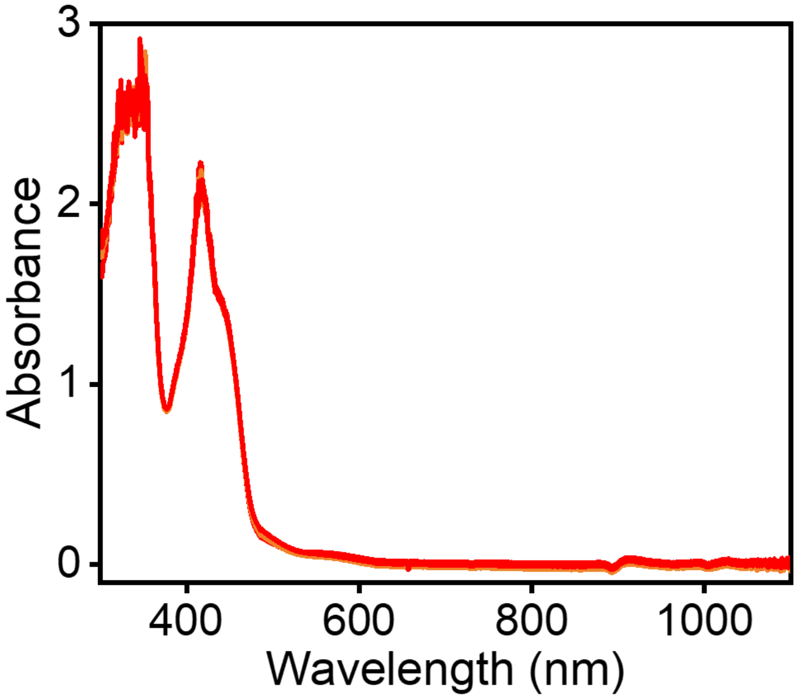

Figure S8. UV-vis absorption depicting no reactivity of 0.27 mM **1** on the addition of 7 eq. *m*CPBA in DMF at -40 °C.

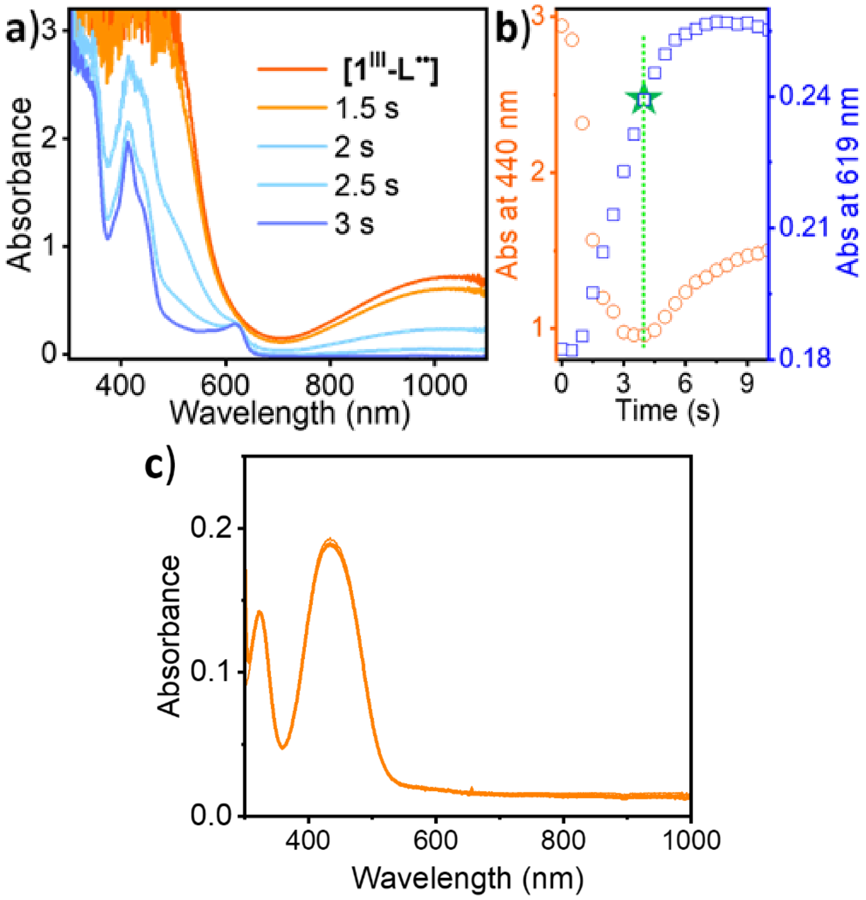

Figure S9. a) UV-Vis absorption depicting changes of **$[1^{III}$-$L^{\bullet\bullet}]$**, on the addition of 3 eq. Fc. *Condition to generate 440 nm species: 0.27 mM **1** + 7 eq. mCPBA in CH_3CN at -40 °C.* b) The corresponding absorption changes at 440 nm and 619 nm. * The green star marks the absorbance of ferrocenium, where all the

440 nm species have been reacted with Fc to form **1**. Ferrocenium 619 nm was found to be 400 M^{-1} cm^{-1} under the conditions employed. c) Reaction of 2 mM Fc with 7 eq. *m*CPBA at -40 °C in CH_3CN did not lead to the formation of ferrocenium.

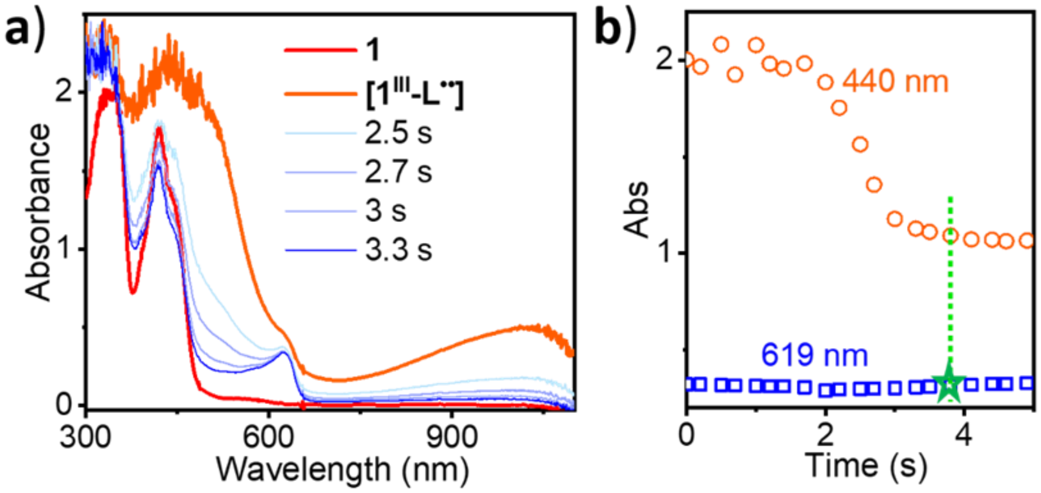

Figure S10. a) UV-Vis absorption depicting changes of 440 nm species, **[1^{III}-L$^{\bullet\bullet}$]** on the addition of 10 eq. Fc. *Condition to generate 440 nm species:* 0.27 mM **1** + 7 eq. *m*CPBA in CH_2Cl_2 at -40 °C generated band at 619 nm due to the formation of ferrocenium. b) The corresponding absorption changes at 440 nm and 619 nm. * The green star marks the absorbance of ferrocenium, where all the 440 nm species have been reacted with Fc to form **1**. Ferrocenium $\varepsilon_{619\ nm}$ was found to be 400 M^{-1} cm^{-1} under the conditions employed

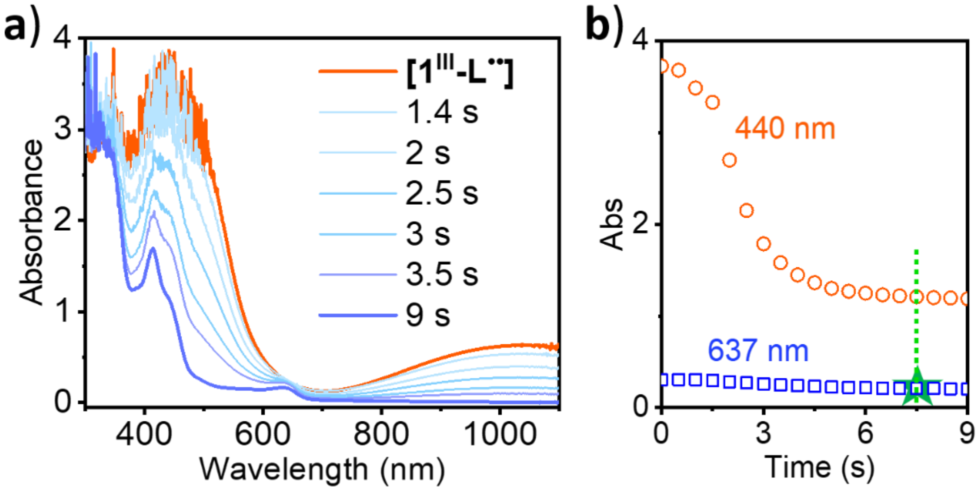

Figure S11. a) UV-Vis absorption depicting changes of **[1^{III}-L$^{\bullet\bullet}$]**, on the addition of 3 eq. AcFc. *Condition to generate 440 nm*: 0.27 mM **1** + 7 eq. *m*CPBA in CH_3CN at -40 °C. b) The corresponding absorption changes at 440 nm and 637 nm. * The green star marks the absorbance of acetyl ferrocenium, where

all the 440 nm species have been reacted with acetyl ferrocene along with the generation of **1**. Acetyl ferrocenium $\varepsilon_{637\ nm}$ was found to be 265 M^{-1} cm^{-1} under the conditions employed.

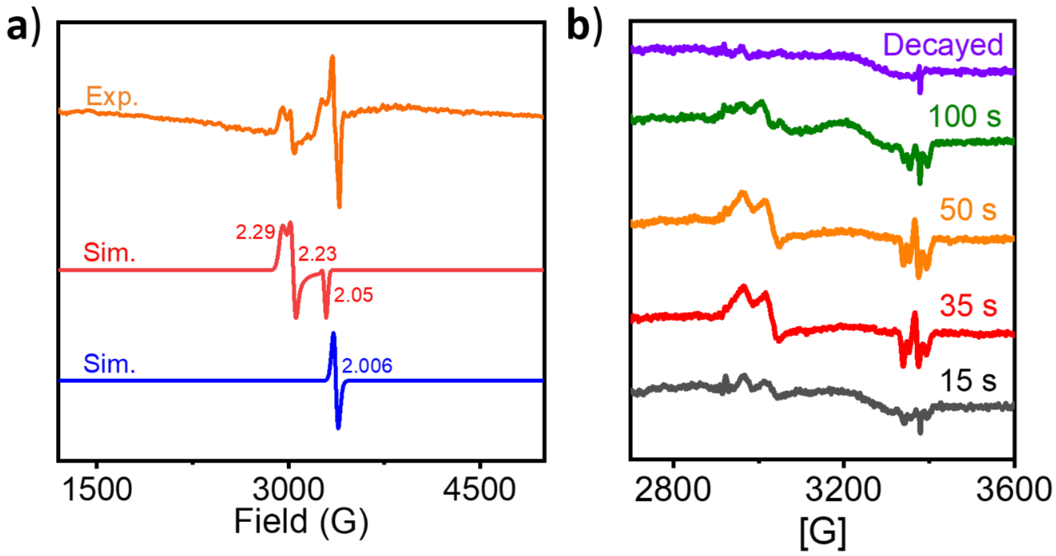

Figure S12. a) X-band EPR of **[1^{III}-L$^{••}$]** in CH_3CN at -150 °C. *Condition to generate 440 nm species:* 0.32 mM **1** + 7 eq. *m*CPBA in CH_3CN at -40 °C. Modulation amplitude 1.98 G; Modulation frequency 100 KHz, and Attenuation 16 dB. *Simulation details*: Red: g_x = 2.29, g_y = 2.23 and g_x = 2.05, g_{avg}= 2.19. Blue: g_{iso} = 2.006. b) Reaction progress for the formation and decay of **[1^{III}-L$^{••}$]** followed by X-band EPR spectroscopy obtained at -150 °C. *Condition to generate 440 nm species:* 2 mM **1** + 7 eq. *m*CPBA in CH_2Cl_2 at -40 °C. Modulation amplitude 1.98 G; Modulation frequency 100 KHz, and Attenuation 16 dB.

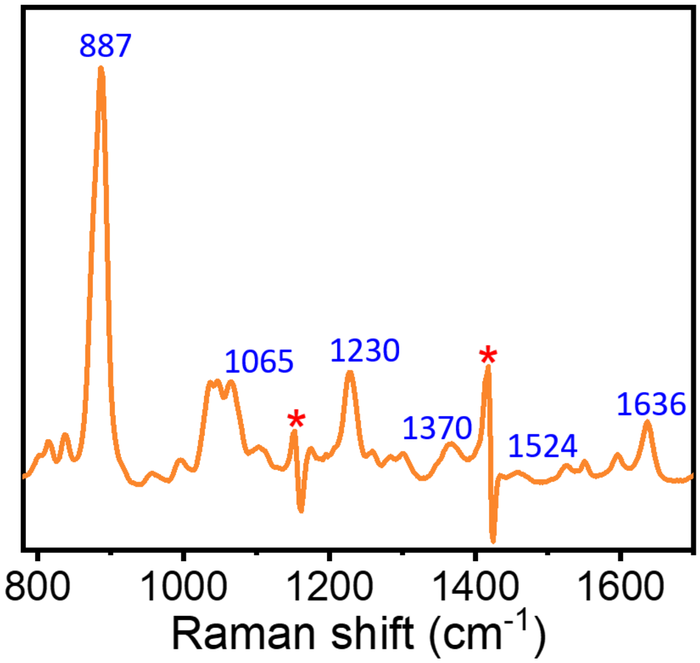

Figure S13. Full-scale resonance Raman spectrum of **[1^{III}-L$^{••}$]** at λ_{exc} 473 nm in CH_2Cl_2, at -80 °C. *Artifacts due to imperfect solvent subtraction. *Condition to generate 440 nm species:* 0.2 mM **1** + 7 eq. *m*CPBA in CH_2Cl_2 at -80 °C.

2. Computational details

3.1 Computational details

All geometries were optimized in Gaussian 16,[2] using the M06-L density functional[3] with the def2-SVP basis set.[4] Weigend's universal fitting basis set[5] was used via the *W06* keyword. The PCM solvation model[6] was used, with acetonitrile specified as the solvent. Frequency analysis confirmed all located stationary points were minima. An *UltraFine* grid was specified, along with increased two-electron integral accuracy (*acc2e=16*). See below for an example input.

Example input line:
#p Integral(acc2e=16,grid=ultrafine) SCRF(PCM,Solvent=Acetonitrile) Pop=Always uM06L/Def2SVP/W06 SCF=XQC Opt(MaxStep=5) Freq=NoRaman

For the three electron oxidized species (with charge = 3+), several structures were considered: with no axial ligand **[1-L]**$^{3+}$; with one or two hydroxy moieties **[1-L-OH]**$^{2+}$ and **[1-L-(OH)$_2$]**$^{1+}$; and with one or two acetonitrile molecules **[1-L-MeCN]**$^{3+}$ and **[1-L-(MeCN)$_2$]**$^{3+}$, all in the axial position(s). All geometries of the three electron oxidized species were optimized in the low spin (LS), intermediate spin (IS) and high spin (HS) states. All converged solutions were tested for stability using the *Stable=Opt* keyword, and the geometries were reoptimized in the case an instability was found. In every 5-coordinate case, the LS state was found to be lowest in energy (energetics discussed further below).

At the optimized (M06-L/def2-SVP/PCM) geometries, single point (SP) calculations were performed in ORCA 5.0.1.[7] These calculations employed the local density functional M06-L,[3a] the hybrid generalized gradient approximation (GGA) PBE0[8], and the hybrid meta-GGA PW6B95.[9] The self-consistent D4 model[10] was used for the calculations with PBE0 and PW6B95. All the SP calculations employed the def2-TZVPP basis set,[4] the cPCM solvation model[11] with acetonitrile specified as the solvent, and the resolution of the identity (RI) approximation to speed up the Coulomb integrals,[12] in combination with Weigend's universal fitting basis set (*def2/J*).[5] The calculations with the hybrid functionals (PBE0 and PW6B95) also employed the *COSX* approximation for the exchange integrals, again with Weigend's universal fitting basis set (*def2/J*).[5] All these calculations used an SCF convergence criterion of 10^{-8} a.u. (*TightSCF*). See below for an example input line. Every method used also found the LS state to be the lowest in energy of the five-coordinate species (Table S1). All orbital isosurfaces were plotted in IboView v2021[13] to enclose 80% of the electron density. The following notation is used to label the IBOs: s(Γ-IBO)^{O}, where s=(α,β) is the spin, Γ=(σ,π,δ) is the symmetry and O=(1,2) is the occupation. All spin density isosurfaces were also rendered with IboView, at +/- 0.004 in green/purple, respectively. Qualitative molecular orbital diagrams for the **LS$_A$** and **LS$_B$** states of **[Ni-MeCN]**$^{3+}$ (Figures S22-23) were made by using the corresponding orbital transformation.[14]

Example input line:
! PW6B95 D4 def2-TZVPP def2/J RIJCOSX DefGrid2 CPCM(Acetonitrile) TightSCF NoTrah

3.2 Structural agreement with experiments

Table S1. Key bond distances (in Å) of **1** were obtained via X-ray diffraction (XRD) and DFT (M06-L/def2-SVP/PCM).

	**Bond distance**			
	**Ni-O$_a$**	**Ni-O$_b$**	**Ni-N$_a$**	**Ni-N$_b$**
**XRD**[1]	1.852	1.852	1.848	1.855
	1.847	1.860	1.852	1.868
**avg.**	1.853		1.856	
**DFT**	1.867		1.865	
deviation	0.014		0.009	

Satisfied with the structural performance of the chosen M06-L/def2-SVP/PCM(Acetonitrile) methodology (Table S1), which we have previously applied to high-valent Nickel species,[15] we proceeded to examine the electronic structure of **1** and its three electron oxidized counterpart, **[1-L]$^{3+}$**, via an Intrinsic Bonding Orbital (IBO) analysis.

3.3 Electronic structure

The formal assignment of **1** as a Ni(II) compound is fully consistent with our calculated electronic structure, obtained via single-point calculations at the optimized geometry with a larger basis set (def2-TZVPP). As expected, **1** exhibit an intrinsic *d*^{8} Ni(II) configuration and dative σ-bonding in the first coordination sphere of the metal center (Figure S14).

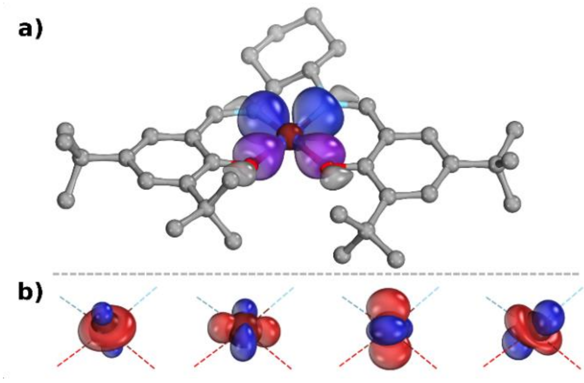

Figure S14. IBO analysis of **1** in the **LS** state: 4 x (σ-IBO)^2 (a) and 4 x (δ-IBO)^2 (b), calculated with M06-L/def2-TZVPP/cPCM//M06-L/def2-SVP/PCM.

The three-electron oxidized complex with no axial ligands **[1-L]$^{3+}$** shows a near intrinsic *d*^{8} configuration (Figure S14), implying a Ni(II) center and a triply oxidized ligand. However, the slight mixing of the π-system into one of the *d*-orbitals (Figure S14) hints at the possibility of metal oxidation. Upon the likely subsequent binding of one or two axial ligands (either hydroxide or acetonitrile), **[1-L-MeCN]$^{3+}$**, **[1-L-(MeCN)$_2$]$^{3+}$**, and **[1-L-OH]$^{2+}$** exhibit intrinsic *d*^{7} configurations (Figure 5 and Figures S15-S17), implying a Ni(III) center and a doubly oxidized ligand, *i.e.*, **[1^{III}-L$^{••}$]**. Despite the observed functional dependence for **[1-L-(OH)$_2$]$^{1+}$** (energetics discussed below), it too shows mixed metal and ligand oxidation in the LS state:

either a Ni(IV) center and a singly oxidized ligand **[1^{IV}-L$^{•}$]** (with M06-L, Figure S18) or a d^7 Ni(III) center with a doubly oxidized ligand **[1^{III}-L$^{••}$]** (with PW6B95-D4, Figure S19). As expected for the d^6 LS state, where three alpha electrons are aligned antiparallel to three beta electrons, reduced spin density is found at the metal center (Figure S20, top) as compared to the d^7 LS state (Figure S20, bottom), which has an unpaired electron at the metal center.

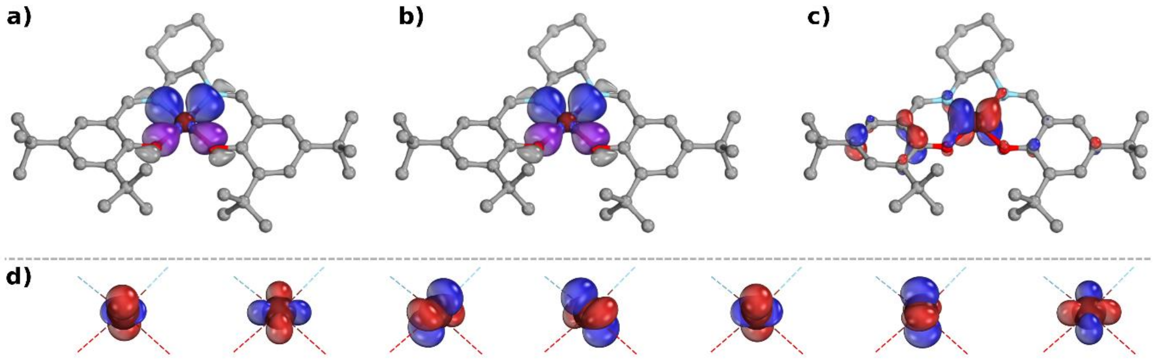

Figure S15. IBO analysis of **[1-L]$^{3+}$** in the **LS** state: 4 x α(σ-IBO)^1 (a), 4 x β(σ-IBO)^1 (b), 1 x Ni-based (δ-IBO)^1, hybridizing with the ligand π-system (c), and 7 x (δ-IBO)^1 calculated with M06-L/def2-TZVPP/cPCM//M06-L/def2-SVP/PCM.

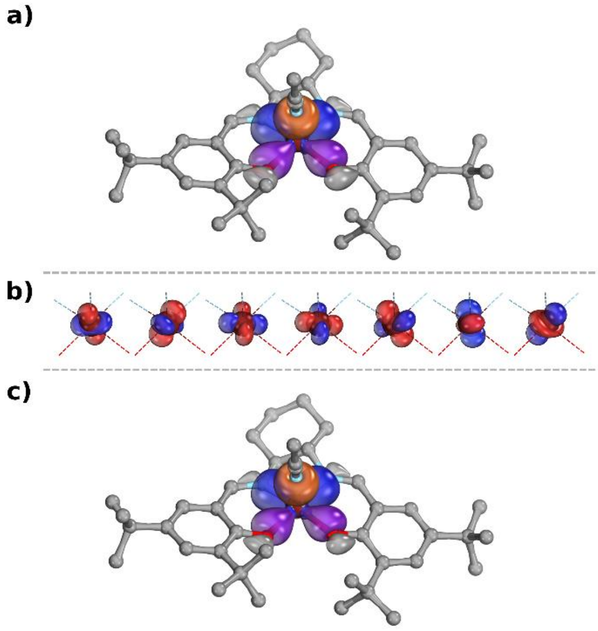

Figure S16. IBO analysis of **[1-L-MeCN]$^{3+}$** in the **LS$_A$** state: 5 x α(σ-IBO)^1 (a), 7 x (δ-IBO)^1 (b), and 5 x β(σ-IBO)^1, calculated with M06-L/def2-TZVPP/cPCM//M06-L/def2-SVP/PCM.

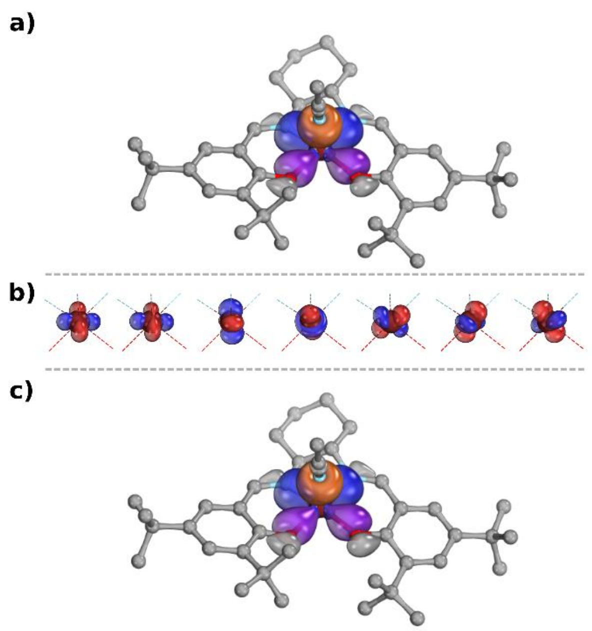

Figure S17. IBO analysis of **[1-L-MeCN]$^{3+}$** in the **LS$_B$** state: 5 x α(σ-IBO)^1 (a), 7 x (δ-IBO)^1 (b), and 5 x β(σ-IBO)^1, calculated with M06-L/def2-TZVPP/cPCM//M06-L/def2-SVP/PCM.

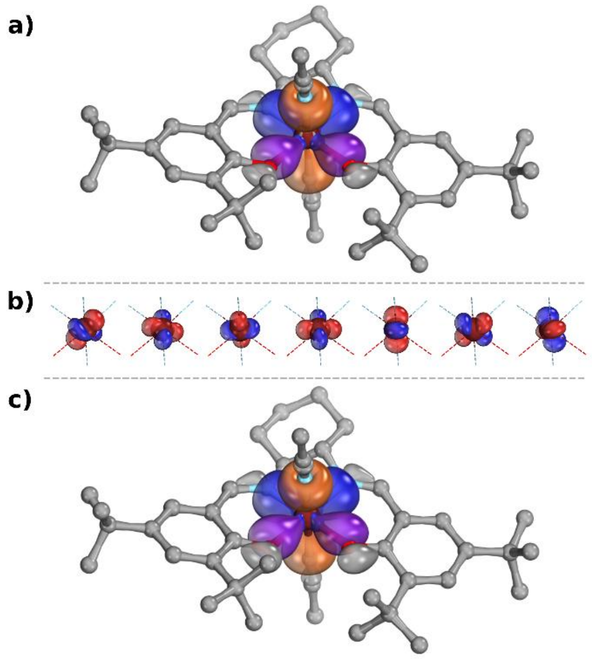

Figure S18. IBO analysis of **[1-L-(MeCN)$_2$]$^{3+}$** in the **LS** state: 6 x α(σ-IBO)^1 (a), 7 x (δ-IBO)^1 (b), and 6 x β(σ-IBO)^1, calculated with M06-L/def2-TZVPP/cPCM//M06-L/def2-SVP/PCM.

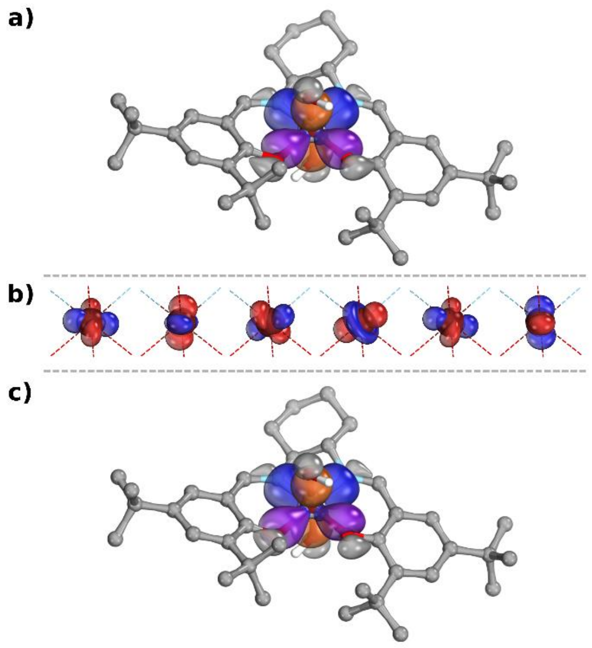

Figure S19. IBO analysis of **$[1\text{-L-(OH)}_2]^{1+}$** in the **LS** state: 6 x $\alpha(\sigma\text{-IBO})^1$ (a), 6 x $(\delta\text{-IBO})^1$ (b), and 6 x $\beta(\sigma\text{-IBO})^1$, calculated with **M06-L**/def2-TZVPP/cPCM//M06-L/def2-SVP/PCM.

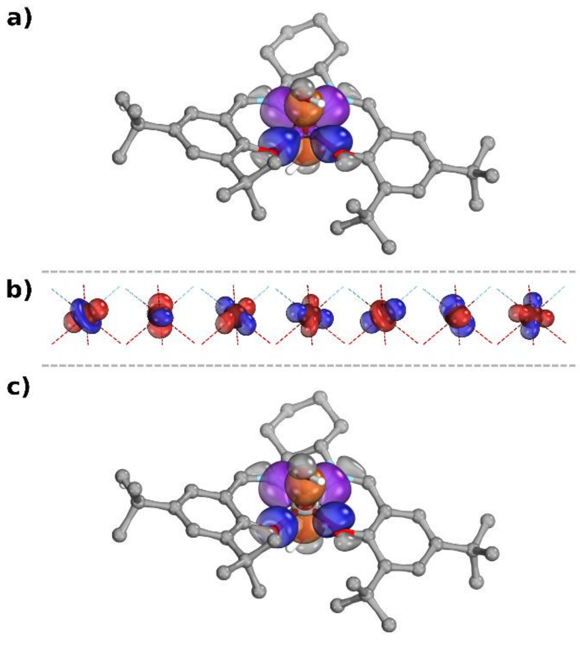

Figure S20. IBO analysis of **$[1\text{-L-(OH)}_2]^{1+}$** in the **LS** state: 6 x $\alpha(\sigma\text{-IBO})^1$ (a), 7 x $(\delta\text{-IBO})^1$ (b), and 6 x $\beta(\sigma\text{-IBO})^1$, calculated with **PW6B95-D4**/def2-TZVPP/cPCM//M06-L/def2-SVP/PCM.

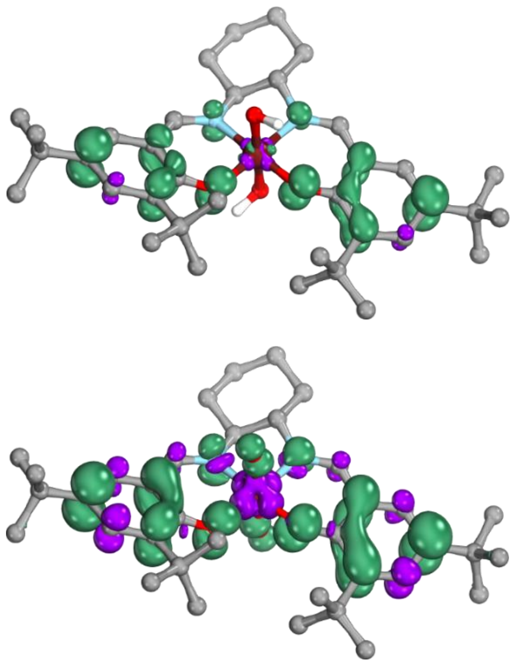

Figure S21. Spin density plots of **[1-L-(OH)$_2$]$^{1+}$**, calculated with **M06-L**/def2-TZVPP/cPCM//M06-L/def2-SVP/PCM (top) and **PW6B95-D4**/def2-TZVPP/cPCM//M06-L/def2-SVP/PCM (bottom). Positive spin density is depicted in green, and negative spin density in purple (isosurface 0.004). Hydrogen atoms are omitted for clarity. Rendered in IboView v2021.[13]

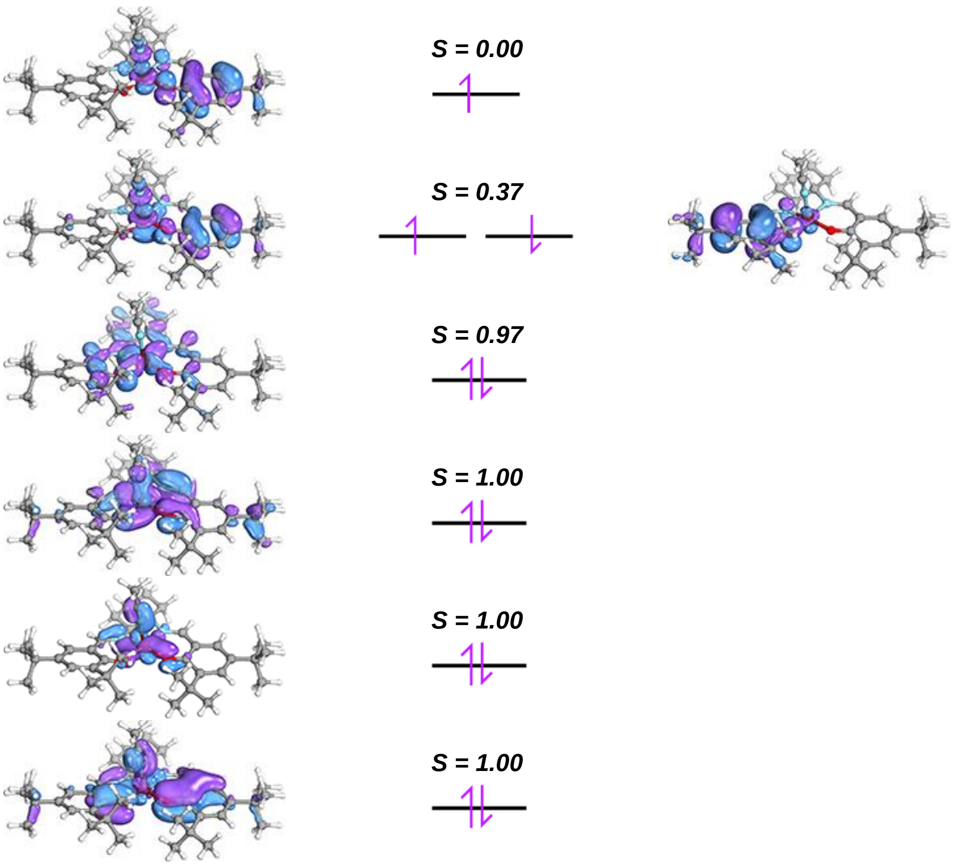

Figure S22: Qualitative molecular orbital diagram of [Ni-L-MeCN]$^{3+}$ in the **LS$_A$** state. The corresponding orbitals[14] and their overlap integrals, *S*. Calculated with M06-L/def2-TZVPP//M06-L/def2-SVP.

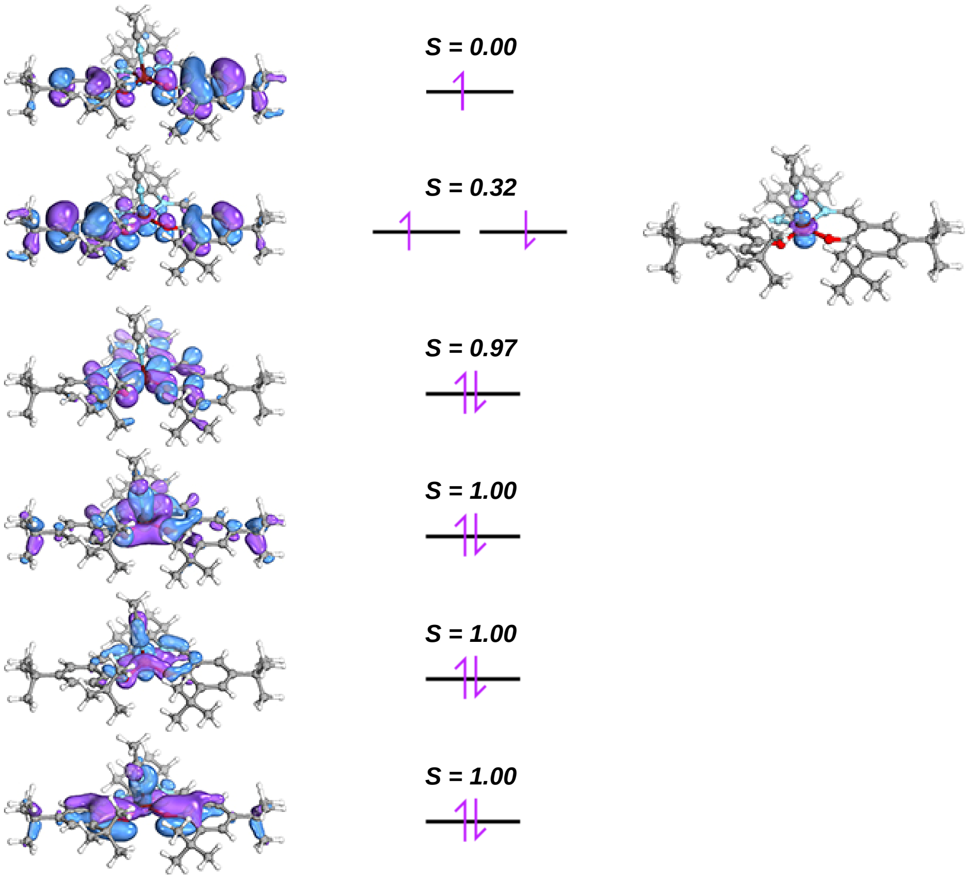

Figure S23: Qualitative molecular orbital diagram of [Ni-L-MeCN]$^{3+}$ in the **LS$_B$** state. The corresponding orbitals[14] and their overlap integrals, *S*. Calculated with M06-L/def2-TZVPP//M06-L/def2-SVP.

4.2.1 Energetics

The LS state is either the favoured ground state for all complexes or near-degenerate with the IS (as in the case of **[1-L-MeCN]$^{3+}$**) with M06-L (Table S2). The other tested functionals (PW6B95-D4 and PBE0-D4) broadly agree with this spin-state ordering, although the magnitude of the separation does change somewhat. One exception is **[1-L-(OH)$_2$]$^{1+}$**, where PW6B95-D4 and PBE0-D4 predict the IS to be favoured over the LS by 5.0 and 5.1 kcal mol^{-1}, respectively. This is, however, inconsistent with the measured EPR signal.

Table S2. Summed electronic energies, E_{SCF}, and zero-point vibrational energies (ZPVE) in kcal mol^{-1} of all modelled species, each relative to the LS (or LS_B, if applicable) state.

**Structure**	**Spin state**	**E_{SCF} + ZPVE (kcal mol^{-1})** Level of theory (SP//Geom)		
		**PW6B95**-D4/def2-TZVPP/cPCM //M06-L/def2-SVP/PCM	**PBE0**-D4/def2-TZVPP/cPCM //M06-L/def2-SVP/PCM	**M06-L**/def2-TZVPP/cPCM //M06-L/def2-SVP/PCM
**1**	**LS**	0.00	0.00	0.00
	**IS**	12.32	10.70	15.56
**[1-L]$^{3+}$**	**LS**	0.00	0.00	0.00
	**IS**	9.54	6.18	12.27
	**HS**	31.63	30.16	35.92
**[1-L-MeCN]$^{3+}$**	**LS_A**	/	/	-0.43
	**LS_B**	0.00	0.00	0.00
	**IS**	1.15	1.11	1.17
	**HS**	24.82	24.11	21.75
**[1-L-(MeCN)$_2$]$^{3+}$**	**LS**	0.00	0.00	0.00
	**IS**	-0.13	-0.21	-0.40
	**HS**	21.02	19.72	17.26
**[1-L-OH]$^{2+}$**	**LS**	0.00	0.00	0.00
	**IS**	3.18	3.52	4.75
	**HS**	9.53	7.79	13.17
**[1-L-(OH)$_2$]$^{1+}$**	**LS**	0.00	0.00	0.00
	**IS**	-4.97	-5.05	7.08
	**HS**	7.01	5.17	15.87

5. Reactivity studies

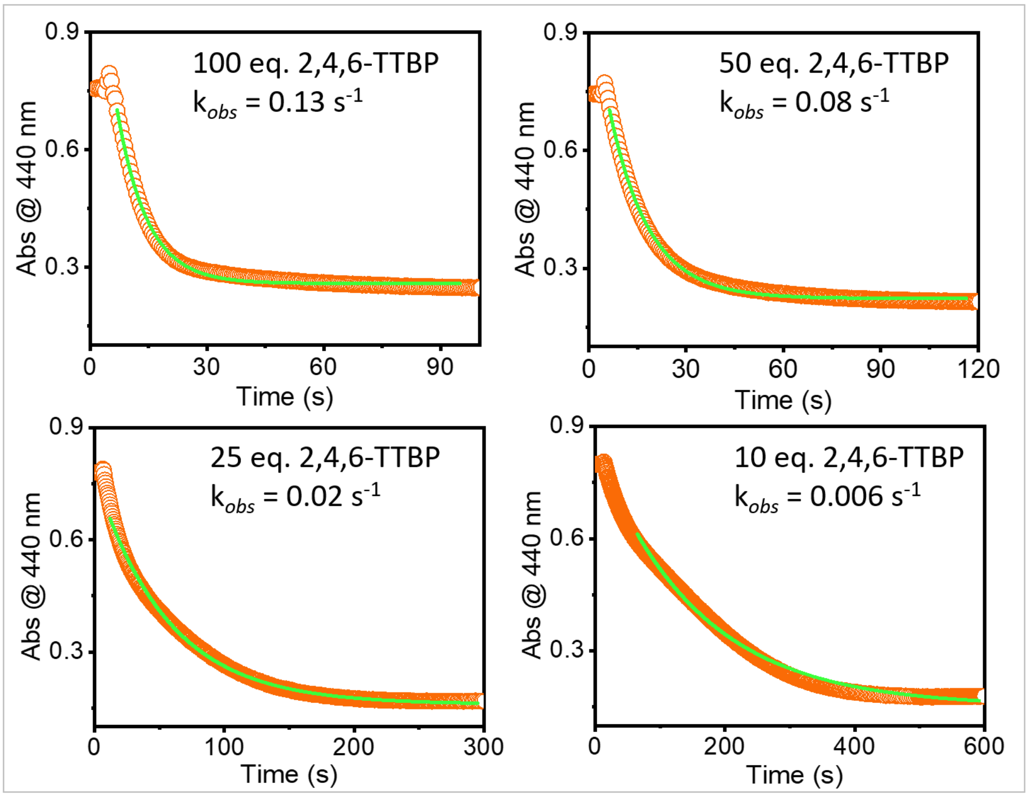

Figure S24: Decay profile of 440 nm species, **[1^{III}-L$^{\bullet\bullet}$]** with different eq. 2,4,6-TTBP and their k_{obs}. *Conditions to generate 440 nm species:* 0.05 mM **1** + 7 eq. *m*CPBA in CH_3CN at -40 °C.

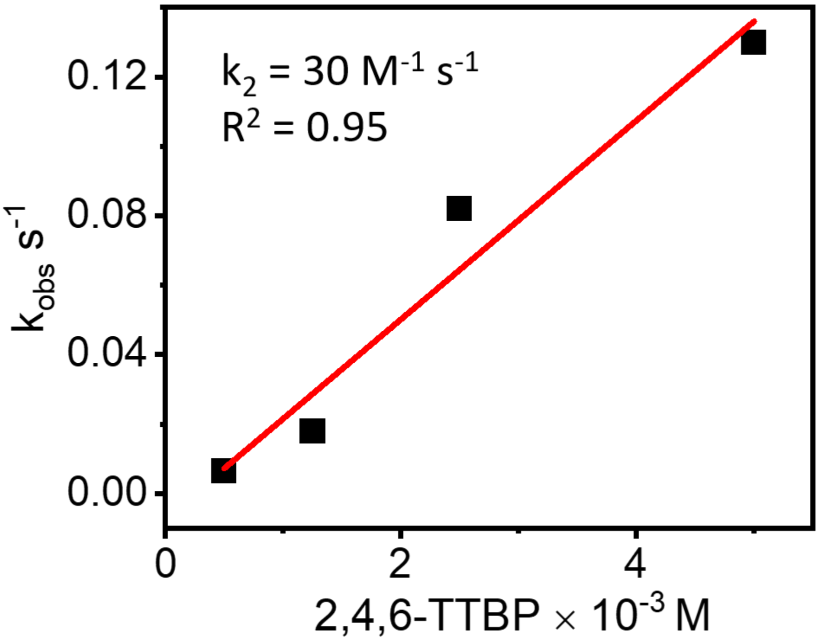

Figure S25: Plot of k_{obs} against the concentration of 2,4,6-TTBP to get the corresponding k_2.

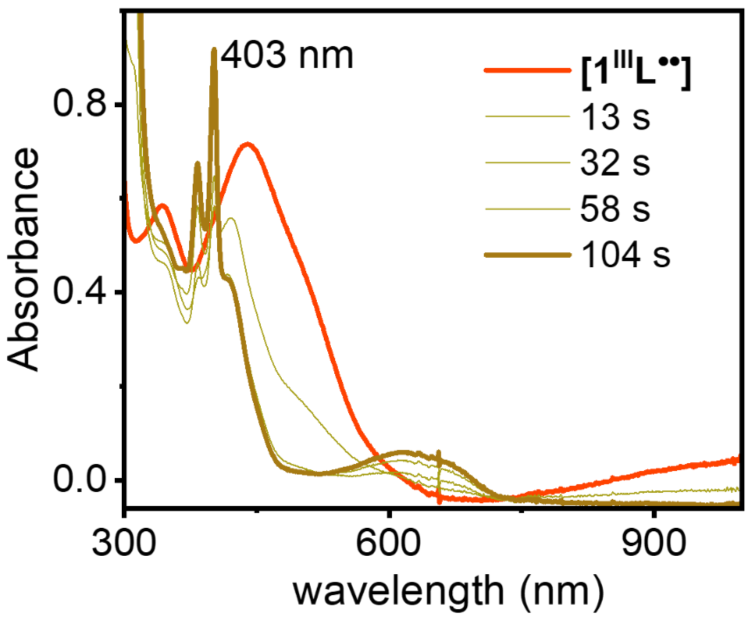

Figure S26: UV-Vis absorption spectral change of **[1^{III}-L$^{••}$]** with 100 eq. 2,4,6-TTBP in CH_3CN at -40 °C. Generation of 2,4,6-tri-tert-butylphenoxyl radical (2,4,6-TTBP$^{•+}$) is observed by the appearance of its characteristics absorption bands at 385 nm, 403 nm, and 626 nm.[16] *Conditions to generate 440 nm species:* 0.05 mM **1** + 7 eq. *m*CPBA in CH_3CN at -40 °C.

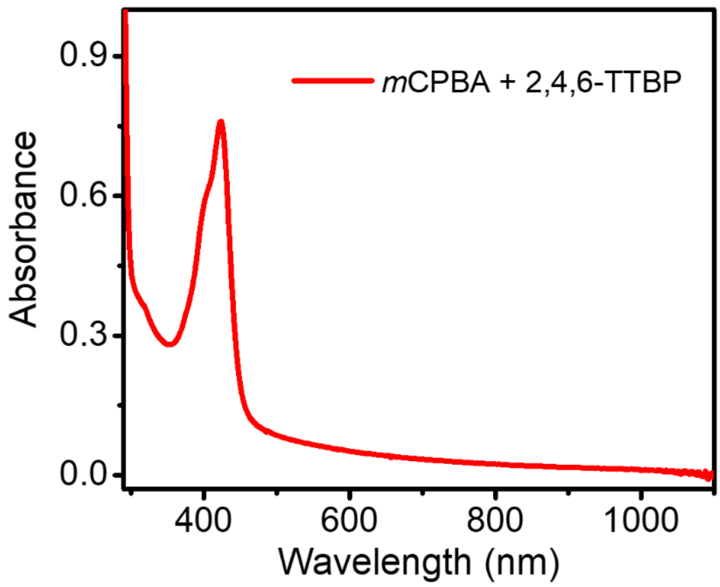

Figure S27: Reaction of *m*CPBA and 2,4,6-TTBP in CH_3CN at RT monitored by UV-Vis absorption spectroscopy.

Note: This reaction did not yield the formation of 2,4,6-tri-tert-butylphenoxyl radical species (2,4,6-TTBP$^{•+}$); see Figure S22.

.

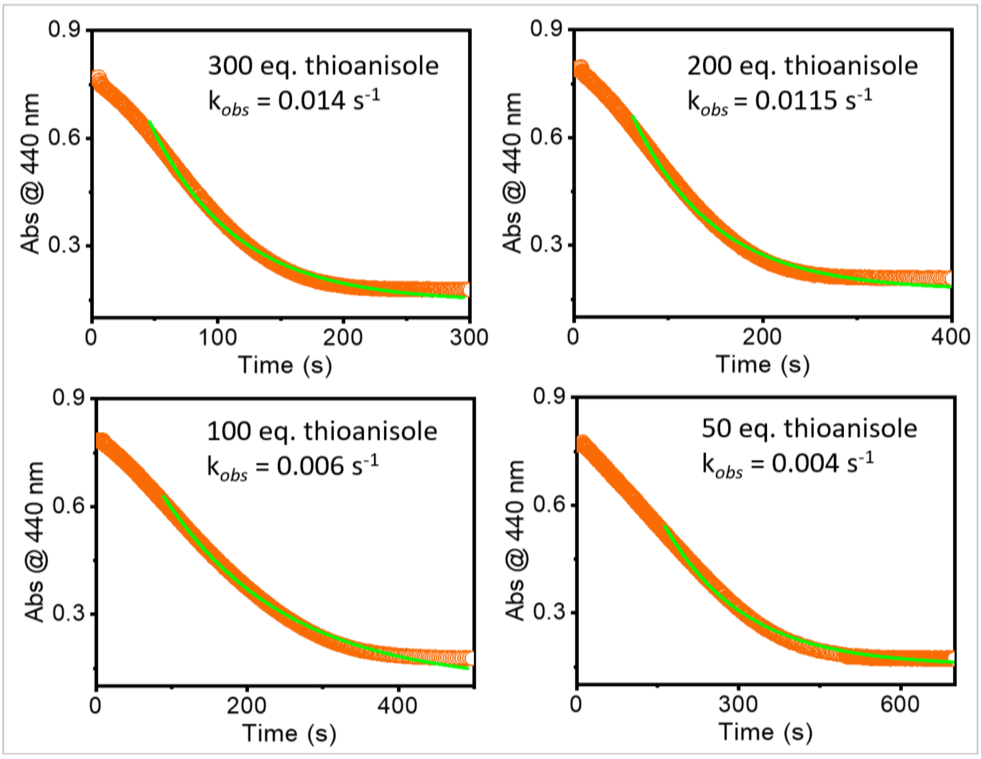

Figure S28: Decay profile of 440 nm species, **[1^{III}-L$^{••}$]** with different eq. thioanisole and their k_{obs}. *Conditions to generate 440 nm species:* 0.05 mM **1** + 7 eq. *m*CPBA in CH_3CN at -40 °C.

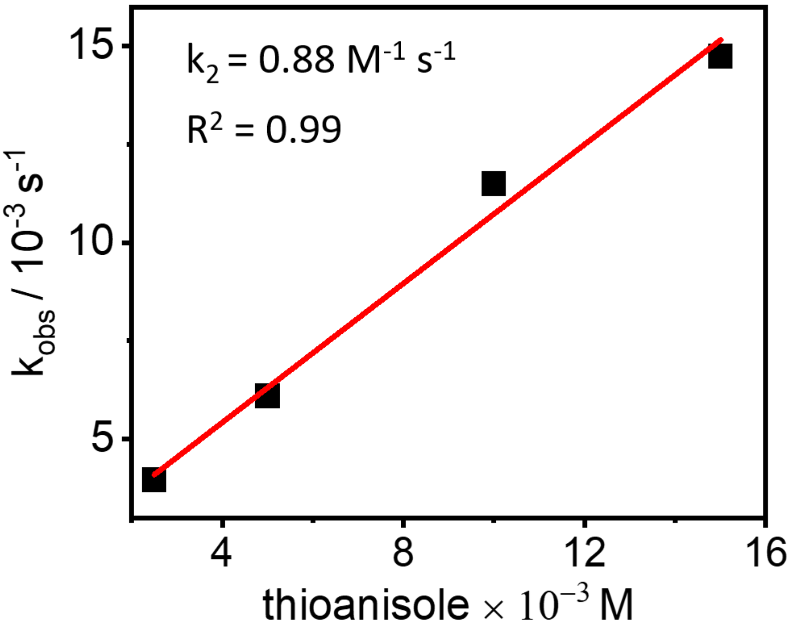

Figure S29: Plot of k_{obs} against the concentration of thioanisole to get the corresponding k_2.

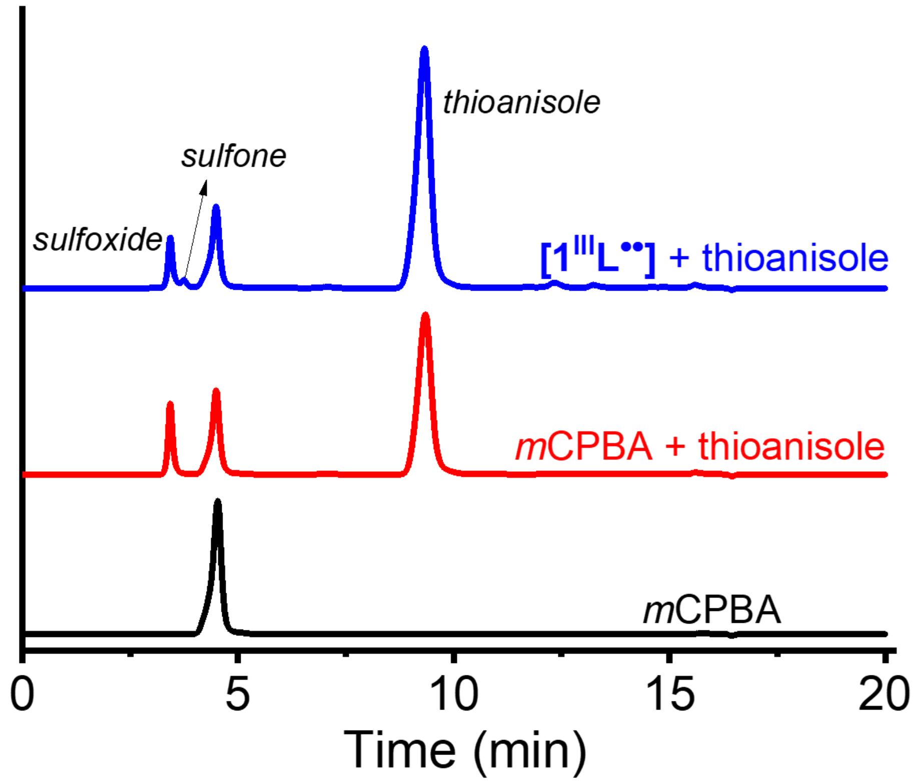

Figure S30: Product analysis of the reaction of **[1^{III}-L$^{••}$]** with 10 eq. thioanisole (blue), 7 eq. *m*CPBA with 10 eq. thioanisole under the same condition (red) and 5 mM mCPBA in CH_3CN by HPLC. *Conditions to generate* **[1^{III}-L$^{••}$]** *:* 0.4 mM **1** + 7 eq. *m*CPBA + 10 eq. thioanisole in CH_3CN at -40 °C.

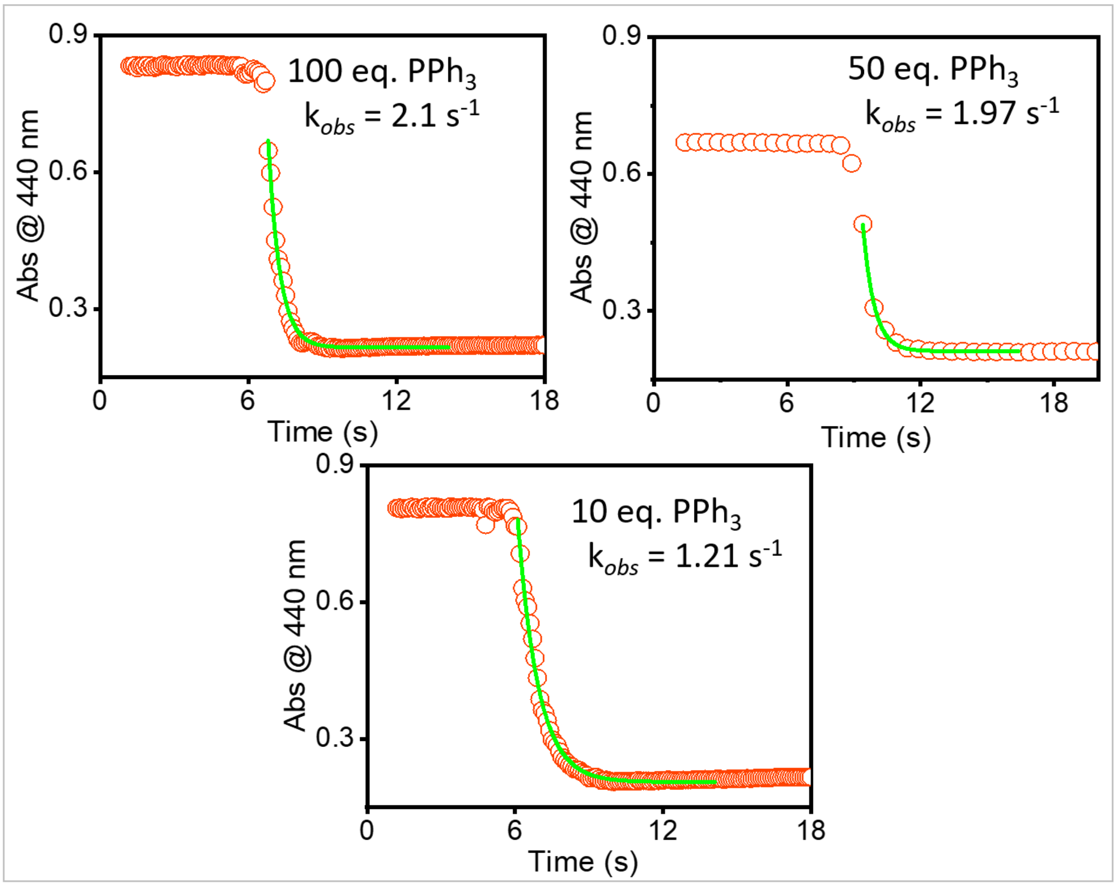

Figure S31: Decay profile of 440 nm species, **[1^{III}-$L^{\bullet\bullet}$]** with different eq. PPh_3 and their k_{obs}. *Conditions to generate 440 nm species:* 0.05 mM **1** + 7 eq. *m*CPBA in CH_3CN at -40 °C.

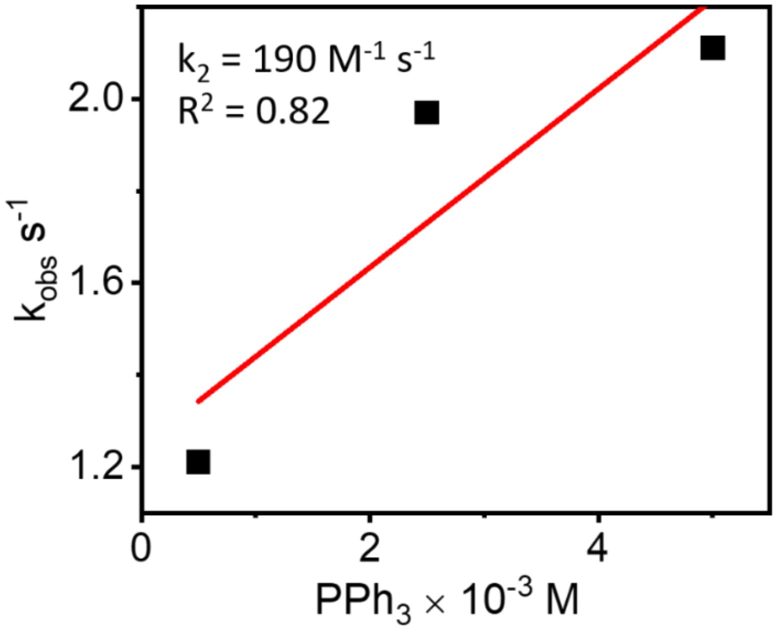

Figure S32: Plot of k_{obs} against the concentration of PPh_3 to get the corresponding k_2.

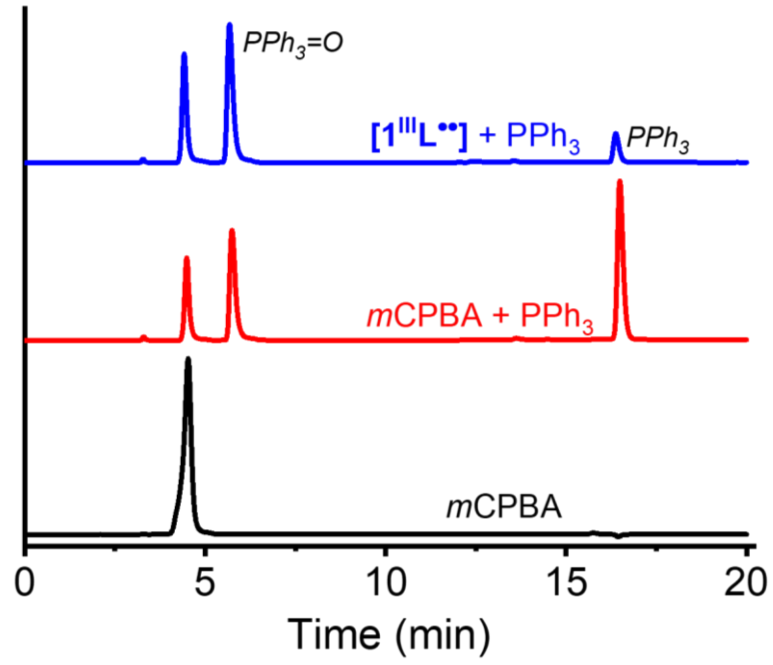

Figure S33: Product analysis of the reaction of **[1^III-L••]** with 10 eq. PPh_3 (blue), *m*CPBA with 10 eq. PPh_3 under the same conditions (red) and 5 mM *m*CPBA (black) in CH_3CN by HPLC.
Conditions to generate **[1^III-L••]** *:* 0.4 mM **1** + 7 eq. *m*CPBA + 10 eq. PPh_3 in CH_3CN at -40 °C.

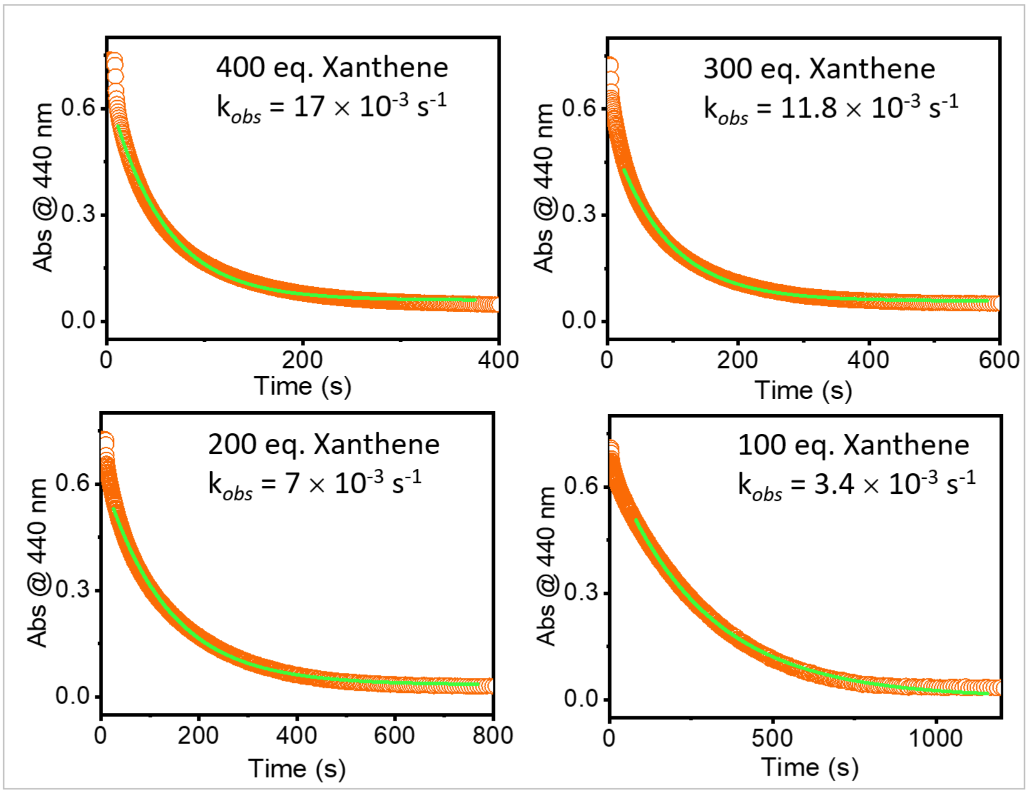

Figure S34: Decay profile of 440 nm species *to* generate 440 nm species, **[1^III-L••]** with different eq. xanthene and their k_{obs}. *Conditions to generate 440 nm species:* 0.05 mM **1** + 7 eq. *m*CPBA in CH_3CN at -40 °C.

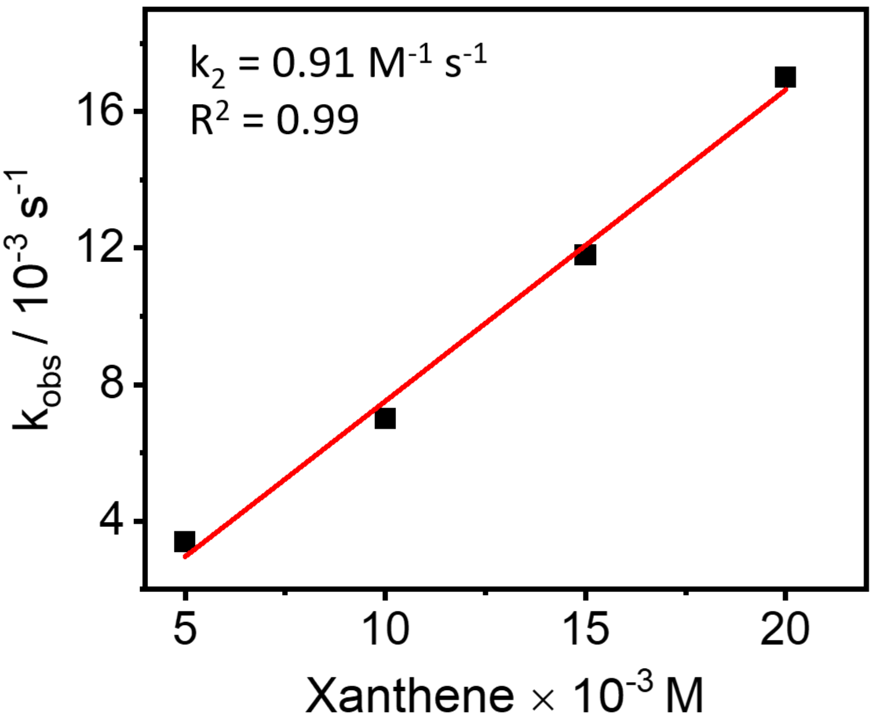

Figure S35: Plot of k$_{obs}$ against the concentration of xanthene to get the corresponding k_2.

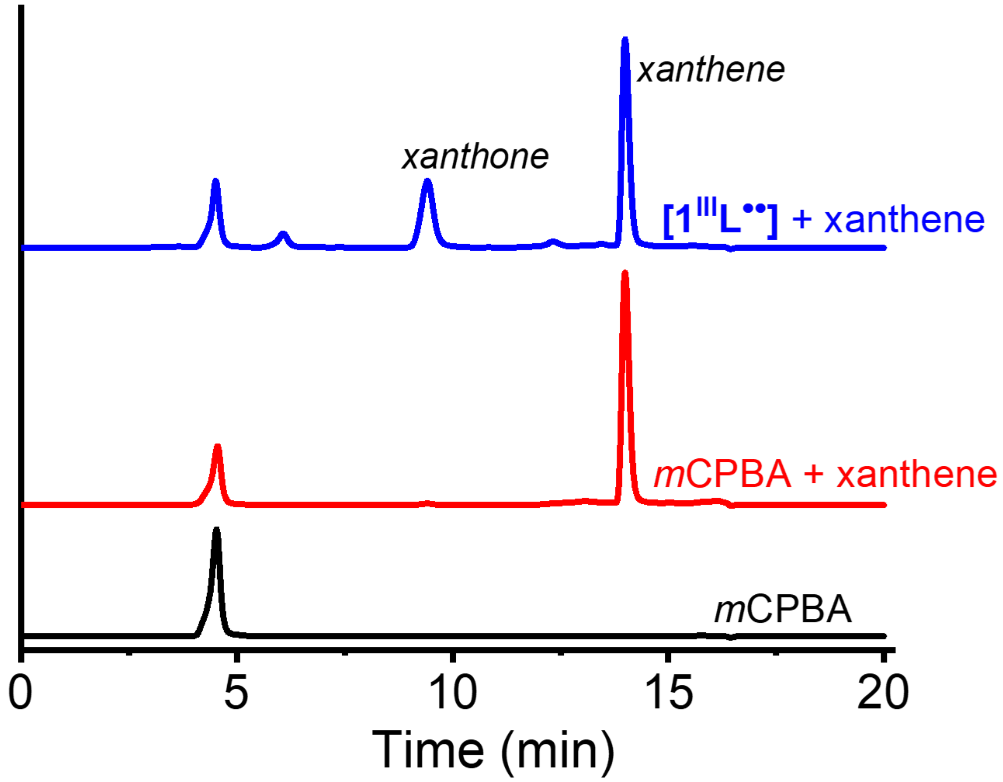

Figure S36: Product analysis of the reaction of **[1^{III}-L$^{••}$]** with 10 eq. xanthene (blue), 7 eq. *m*CPBA with 10 eq. xanthene under the same conditions (red) and 5 mM *m*CPBA (black) in CH_3CN by HPLC. *Conditions to generate* **[1^{III}-L$^{••}$]***:* 0.4 mM **1** + 7 eq. *m*CPBA + 10 eq. xanthene in CH_3CN at -40 °C.

References

[1] Y. Shimazaki, F. Tani, K. Fukui, Y. Naruta, O. Yamauchi, *J. Am. Chem. Soc.* **2003**, *125*, 10512–10513.

[2] *Gaussian 16 Rev. B.01* M. J. Frisch, G. W. Trucks, H. B. Schlegel, G. E. Scuseria, M. A. Robb, J. R. Cheeseman, G. Scalmani, V. Barone, G. A. Petersson, H. Nakatsuji, X. Li, M. Caricato, A. V. Marenich, J. Bloino, B. G. Janesko, R. Gomperts, B. Mennucci, H. P. Hratchian, J. V. Ortiz, A. F. Izmaylov, J. L. Sonnenberg, Williams, F. Ding, F. Lipparini, F. Egidi, J. Goings, B. Peng, A. Petrone, T. Henderson, D. Ranasinghe, V. G. Zakrzewski, J. Gao, N. Rega, G. Zheng, W. Liang, M. Hada, M. Ehara, K. Toyota, R. Fukuda, J. Hasegawa, M. Ishida, T. Nakajima, Y. Honda, O. Kitao, H. Nakai, T. Vreven, K. Throssell, J. A. Montgomery Jr., J. E. Peralta, F. Ogliaro, M. J. Bearpark, J. J. Heyd, E. N. Brothers, K. N. Kudin, V. N. Staroverov, T. A. Keith, R. Kobayashi, J. Normand, K. Raghavachari, A. P. Rendell, J. C. Burant, S. S. Iyengar, J. Tomasi, M. Cossi, J. M. Millam, M. Klene, C. Adamo, R. Cammi, J. W. Ochterski, R. L. Martin, K. Morokuma, O. Farkas, J. B. Foresman, D. J. Fox, *Series* **2016**.

[3] a) Y. Zhao, D. G. Truhlar, *Theor. Chem. Acc.* **2008**, *120*, 215-241; b) Y. Zhao, D. G. Truhlar, *J. Chem.Phys.* **2006**, *125*, 194101.

[4] F. Weigend, R. Ahlrichs, *Phys. Chem. Chem. Phys.* **2005**, *7*, 3297-3305.

[5] F. Weigend, *Phys. Chem. Chem. Phys.* **2006**, *8*, 1057.

[6] a) S. Miertuš, E. Scrocco, J. Tomasi, *Chem. Phys.* **1981**, *55*, 117-129; b) S. Miertuš, J. Tomasi, *Chem. Phys.* **1982**, *65*, 239-245; c) J. L. Pascual-ahuir, E. Silla, I. Tuñon, *J. Comput. Chem.* **1994**, *15*, 1127-1138.

[7] a) F. Neese, *WIREs: Comput. Mol. Sci.* **2012**, *2*, 73-78; b) F. Neese, *WIREs: Comput. Mol. Sci.* **2018**, *8*, e1327.

[8] C. Adamo, M. Cossi, V. Barone, *J. Mol. Struct.: THEOCHEM* **1999**, *493*, 145-157.

[9] Y. Zhao, D. G. Truhlar, *J. Phys. Chem. A* **2005**, *109*, 5656-5667.

[10] E. Caldeweyher, S. Ehlert, A. Hansen, H. Neugebauer, S. Spicher, C. Bannwarth, S. Grimme, *J. Chem. Phys.* **2019**, *150*, 154122.

[11] a) V. Barone, M. Cossi, *J. Phys. Chem. A* **1998**, *102*, 1995-2001; b) A. W. Lange, J. M. Herbert, *J. Chem. Phys.* **2010**, *133*, 244111.

[12] F. Neese, *J. Comput. Chem.* **2003**, *24*, 1740-1747.

[13] G. Knizia, *J. Chem. Theory Comput.* **2013**, *9*, 4834-4843.

[14] F. Neese, *J. Phys. Chem. Solids*, 2004, **65**, 781-785

[15] J. S. Steen, G. Knizia, J. E. M. N. Klein, *Angew. Chem. Int. Ed.* **2019**, *58*, 13133-13139; *Angew. Chem.* **2019**, *131*,13267–13273.

[16] T. Wu, S. N. MacMillan, K. Rajabimoghadam, M. A. Siegler, K. M. Lancaster, I. Garcia-Bosch, *J. Am. Chem. Soc.* **2020**, *142*, 12265-12276.
